# Coronavirus envelope protein: current knowledge

**DOI:** 10.1186/s12985-019-1182-0

**Published:** 2019-05-27

**Authors:** Dewald Schoeman, Burtram C. Fielding

**Affiliations:** Molecular Biology and Virology Research Laboratory, Department of Medical Biosciences, University of the Western Cape, Cape Town, South Africa

**Keywords:** Coronavirus, Envelope protein, Topology, Assembly, Budding, Viroporin

## Abstract

**Background:**

Coronaviruses (CoVs) primarily cause enzootic infections in birds and mammals but, in the last few decades, have shown to be capable of infecting humans as well. The outbreak of severe acute respiratory syndrome (SARS) in 2003 and, more recently, Middle-East respiratory syndrome (MERS) has demonstrated the lethality of CoVs when they cross the species barrier and infect humans. A renewed interest in coronaviral research has led to the discovery of several novel human CoVs and since then much progress has been made in understanding the CoV life cycle. The CoV envelope (E) protein is a small, integral membrane protein involved in several aspects of the virus’ life cycle, such as assembly, budding, envelope formation, and pathogenesis. Recent studies have expanded on its structural motifs and topology, its functions as an ion-channelling viroporin, and its interactions with both other CoV proteins and host cell proteins.

**Main body:**

This review aims to establish the current knowledge on CoV E by highlighting the recent progress that has been made and comparing it to previous knowledge. It also compares E to other viral proteins of a similar nature to speculate the relevance of these new findings. Good progress has been made but much still remains unknown and this review has identified some gaps in the current knowledge and made suggestions for consideration in future research.

**Conclusions:**

The most progress has been made on SARS-CoV E, highlighting specific structural requirements for its functions in the CoV life cycle as well as mechanisms behind its pathogenesis. Data shows that E is involved in critical aspects of the viral life cycle and that CoVs lacking E make promising vaccine candidates. The high mortality rate of certain CoVs, along with their ease of transmission, underpins the need for more research into CoV molecular biology which can aid in the production of effective anti-coronaviral agents for both human CoVs and enzootic CoVs.

## Background

Coronaviruses (CoVs) (order *Nidovirales*, family *Coronaviridae*, subfamily *Coronavirinae*) are enveloped viruses with a positive sense, single-stranded RNA genome. With genome sizes ranging from 26 to 32 kilobases (kb) in length, CoVs have the largest genomes for RNA viruses. Based on genetic and antigenic criteria, CoVs have been organised into three groups: α-CoVs, β-CoVs, and γ-CoVs (Table 1) [1, 2]. Coronaviruses primarily infect birds and mammals, causing a variety of lethal diseases that particularly impact the farming industry [3, 4]. They can also infect humans and cause disease to varying degrees, from upper respiratory tract infections (URTIs) resembling the common cold, to lower respiratory tract infections (LRTIs) such as bronchitis, pneumonia, and even severe acute respiratory syndrome (SARS) [5–14]. In recent years, it has become increasingly evident that human CoVs (HCoVs) are implicated in both URTIs and LRTIs, validating the importance of coronaviral research as agents of severe respiratory illnesses [7, 9, 15–17].

**Table 1:** Organisation of CoV species (adapted from Jimenez-Guardeño, Nieto-Torres [18])

Group	Species
α-CoVs	Transmissible gastroenteritis coronavirus (TGEV)
	Canine coronavirus (CCoV)
	Porcine respiratory coronavirus (PRCoV)
	Feline coronavirus (FeCoV)
	Porcine epidemic diarrhoea coronavirus (PEDV)
	Human coronavirus 229E (HCoV-229E)
	Human coronavirus NL63 (HCoV-NL63)
β-CoVs	Bat coronavirus (BCoV)
	Porcine hemagglutinating encephalomyelitis virus (HEV)
	Murine hepatitis virus (MHV)
	Human coronavirus 4408 (HCoV-4408)
	Human coronavirus OC43 (HCoV-OC43)
	Human coronavirus HKU1 (HCoV-HKU1)
	Severe acute respiratory syndrome coronavirus (SARS-CoV)
	Middle Eastern respiratory syndrome coronavirus (MERS-CoV)
γ-CoVs	Avian infectious bronchitis virus (IBV)
	Turkey coronavirus (TCoV)

Some CoVs were originally found as enzootic infections, limited only to their natural animal hosts, but have crossed the animal-human species barrier and progressed to establish zoonotic diseases in humans [19–23]. Accordingly, these cross-species barrier jumps allowed CoVs like the SARS-CoV and Middle Eastern respiratory syndrome (MERS)-CoV to manifest as virulent human viruses. The consequent outbreak of SARS in 2003 led to a near pandemic with 8096 cases and 774 deaths reported worldwide, resulting in a fatality rate of 9.6% [24]. Since the outbreak of MERS in April 2012 up until October 2018, 2229 laboratory-confirmed cases have been reported globally, including 791 associated deaths with a case-fatality rate of 35.5% [25]. Clearly, the seriousness of these infections and the lack of effective, licensed treatments for CoV infections underpin the need for a more detailed and comprehensive understanding of coronaviral molecular biology, with a specific focus on both their structural proteins as well as their accessory proteins [26–30]. Live, attenuated vaccines and fusion inhibitors have proven promising, but both also require an intimate knowledge of CoV molecular biology [29, 31–36].

The coronaviral genome encodes four major structural proteins: the spike (S) protein, nucleocapsid (N) protein, membrane (M) protein, and the envelope (E) protein, all of which are required to produce a structurally complete viral particle [29, 37, 38]. More recently, however, it has become clear that some CoVs do not require the full ensemble of structural proteins to form a complete, infectious virion, suggesting that some structural proteins might be dispensable or that these CoVs might encode additional proteins with overlapping compensatory functions [35, 37, 39–42]. Individually, each protein primarily plays a role in the structure of the virus particle, but they are also involved in other aspects of the replication cycle. The S protein mediates attachment of the virus to the host cell surface receptors and subsequent fusion between the viral and host cell membranes to facilitate viral entry into the host cell [42–44]. In some CoVs, the expression of S at the cell membrane can also mediate cell-cell fusion between infected and adjacent, uninfected cells. This formation of giant, multinucleated cells, or syncytia, has been proposed as a strategy to allow direct spreading of the virus between cells, subverting virus-neutralising antibodies [45–47].

Unlike the other major structural proteins, N is the only protein that functions primarily to bind to the CoV RNA genome, making up the nucleocapsid [48]. Although N is largely involved in processes relating to the viral genome, it is also involved in other aspects of the CoV replication cycle and the host cellular response to viral infection [49]. Interestingly, localisation of N to the endoplasmic reticulum (ER)-Golgi region has proposed a function for it in assembly and budding [50, 51]. However, transient expression of N was shown to substantially increase the production of virus-like particles (VLPs) in some CoVs, suggesting that it might not be required for envelope formation, but for complete virion formation instead [41, 42, 52, 53].

The M protein is the most abundant structural protein and defines the shape of the viral envelope [54]. It is also regarded as the central organiser of CoV assembly, interacting with all other major coronaviral structural proteins [29]. Homotypic interactions between the M proteins are the major driving force behind virion envelope formation but, alone, is not sufficient for virion formation [54–56]. Interaction of S with M is necessary for retention of S in the ER-Golgi intermediate compartment (ERGIC)/Golgi complex and its incorporation into new virions, but dispensable for the assembly process [37, 45, 57]. Binding of M to N stabilises the nucleocapsid (N protein-RNA complex), as well as the internal core of virions, and, ultimately, promotes completion of viral assembly [45, 58, 59]. Together, M and E make up the viral envelope and their interaction is sufficient for the production and release of VLPs [37, 60–64].

The E protein is the smallest of the major structural proteins, but also the most enigmatic. During the replication cycle, E is abundantly expressed inside the infected cell, but only a small portion is incorporated into the virion envelope [65]. The majority of the protein is localised at the site of intracellular trafficking, viz. the ER, Golgi, and ERGIC, where it participates in CoV assembly and budding [66]. Recombinant CoVs have lacking E exhibit significantly reduced viral titres, crippled viral maturation, or yield propagation incompetent progeny, demonstrating the importance of E in virus production and maturation [35, 39, 40, 67, 68].

## Main text

### The envelope protein

#### Structure

**Fig. 1:**
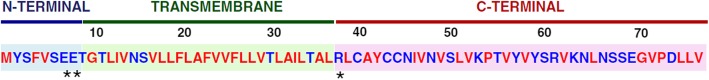
Amino Acid Sequence and Domains of the SARS-CoV E Protein. The SARS-CoV E protein consists of three domains, i.e. the amino (N)-terminal domain, the transmembrane domain (TMD), and the carboxy (C)-terminal domain. Amino acid properties are indicated: hydrophobic (red), hydrophilic (blue), polar, charged (asterisks) [78]

The CoV E protein is a short, integral membrane protein of 76–109 amino acids, ranging from 8.4 to 12 kDa in size [69–71]. The primary and secondary structure reveals that E has a short, hydrophilic amino terminus consisting of 7–12 amino acids, followed by a large hydrophobic transmembrane domain (TMD) of 25 amino acids, and ends with a long, hydrophilic carboxyl terminus, which comprises the majority of the protein (Fig. 1) [1, 60, 72–75]. The hydrophobic region of the TMD contains at least one predicted amphipathic α-helix that oligomerizes to form an ion-conductive pore in membranes [76–78].

Comparative and phylogenetic analysis of SARS-CoV E revealed that a substantial portion of the TMD consists of the two nonpolar, neutral amino acids, valine and leucine, lending a strong hydrophobicity to the E protein [79]. The peptide exhibits an overall net charge of zero, the middle region being uncharged and flanked on one side by the negatively charged amino (N)-terminus, and, on the other side, the carboxy (C)-terminus of variable charge. The C-terminus also exhibits some hydrophobicity but less than the TMD due to the presence of a cluster of basic, positively charged amino acids [80]. Computational predictions regarding the secondary structure of E suggest that the C-terminus of β- and γ-CoVs also contains a conserved proline residue centred in a β-coil-β motif [72]. This motif likely functions as a Golgi-complex targeting signal as mutation of this conserved proline was sufficient to disrupt the localization of a mutant chimeric protein to the Golgi complex and instead localized the protein to the plasma membrane [81].

The SARS-CoV E protein has recently been found to contain a binding motif known as the postsynaptic density protein 95 (PSD95)/*Drosophila* disc large tumour suppressor (Dlg1)/zonula occludens-1 protein (zo-1) (PDZ)-binding motif (PBM), located in the last four amino acids of the C terminus [82]. The PDZ domain is a protein-protein interaction module that can bind to the C-terminus of target proteins such as the cellular adapter proteins involved in host-cell processes important for viral infection [83–86]. Some interaction partners capable of binding to the PBM of SARS-CoV E have been identified and appears to be involved in the pathogenesis of SARS-CoV [18, 66, 82, 87].

The importance of the PBM domain was recently demonstrated in SARS-CoV-infected cells [88]. The PBM domain was either mutated or deleted but reverted to a pathogenic state after several passages in Vero E6 host cells. Deletion of either the last nine resides of SARS-CoV E (ΔPBM) or mutation of the four PBM residues to glycine (mutPBM) resulted in the acquisition of a PBM at the C-terminus of E that was similar to the original PBM sequence. Deleting the last 12 residues of E (Δ6), including the PBM, caused viruses to acquire an alternative PBM different from the sequence of the original PBM. Of particular interest is the mutation of only two of the PBM residues to alanine (altPBM) as these mutants maintained the same mutated sequence after serial passage of infected cells. This suggests that, at least for SARS-CoV E, some minor PBM mutations appear to be tolerated but that a reasonably intact PBM domain is still necessary to avoid revertant mutants [34, 88]. It would be interesting to see if any of these serially passaged PBM mutants are still capable of host cell protein interaction and whether the mutations allow the virus to retain its pathogenicity in both in vivo and in vitro systems. This would prove valuable for the design of a live, attenuated vaccine with a PBM sufficiently mutated to remain intact, but also enough to be non-functional and abolish the pathogenicity of the virus.

#### Localisation

Coronaviruses are distinct from other well-studied enveloped viruses in that they bud into the ERGIC, from where they acquire their membrane envelope [89]. Once in the lumen of the ERGIC, infectious virions make their way through the host secretory pathway to, ultimately, be released from the infected cell [90]. Accordingly, the E protein is localized mainly to the ER and Golgi-complex where it participates in the assembly, budding, and intracellular trafficking of infectious virions [56, 66, 71, 91]. Concern has been raised over the possibility of epitope-tagged E proteins affecting its localisation, but both FLAG-tagged and untagged versions of SARS-CoV E demonstrate this distribution pattern [73, 81, 92]. Nieto-Torres, DeDiego [66] also investigated the subcellular localization of the SARS-CoV E protein using both transfected cells and infected cells and found that in both groups of cells E accumulated at the ER-Golgi, suggesting that the presence of the tag on E did not affect its localization. The authors also reported that the other viral structural proteins did not appear to significantly influence the localization of the E protein, concluding that localization of SARS-CoV E occurs at the ERGIC, whether expressed alone or during an infection. Although studies investigating the localisation of E have only used FLAG-tagged versions of the protein, the results suggest that epitope tags do not appear to have any significant influence on the localisation of the CoV E protein to the ER-Golgi region. However, there is no evidence to support whether the presence of larger epitope-tags, such as glutathione S-transferase (GST) and green-fluorescent protein (GFP), might interfere with CoV E protein localisation.

Establishing which part of the E protein contains the information responsible for targeting to the ERGIC is important as it might allude to how CoVs interact with both other viral proteins and host proteins to facilitate the assembly of new infectious viral progeny. However, research into this aspect has been sparse. Using SignalP, Wu, Zhang [79] reported a predicted signal peptide cleavage site at the N-terminus of the SARS-CoV E protein. However, Raamsman, Locker [71] reported no difference in the electrophoretic mobility of the mouse hepatitis virus (MHV) A59 E protein during or after its membrane integration and concluded that MHV E has no cleavable signal peptide sequence. Corse and Machamer [93] were the first to identify that the C-terminus of the IBV E protein housed the Golgi-targeting information. They explored the possibility of a targeting signal located in the luminal N-terminus but found the truncated terminus to be transported to the cell surface. Conversely, truncation of the C-terminus and production of a chimeric E protein both demonstrated retention at the Golgi complex, leading the authors to conclude that the Golgi-targeting information of the IBV E protein was located in its C-terminus. Further truncation of the C-terminus narrowed down the bulk of the targeting information to a sequence motif located between amino acid residues 44 and 72.

Building on this, Cohen, Lin [81] found that the Golgi complex-targeting information of the SARS-CoV E protein was also located in the C-terminus. The authors specifically demonstrated that neither the mutation of a highly conserved proline residue nor the disruption of the predicted β-strands, that stabilise the β-hairpin on either side of the conserved proline residue, were sufficient to disrupt the targeting of the SARS-CoV E protein to the Golgi complex. Using an N-terminus chimeric protein, the authors went on to investigate the possibility of Golgi-targeting information present in the E protein N-terminus. Interestingly, the N-terminus chimaera was targeted to the Golgi region and the authors concluded that the N-terminus of the SARS-CoV E protein contains additional targeting information. They further remarked that the existence of targeting information in both the N- and C-terminus likely explains why the localization of full-length E proteins with mutations only in the C-terminus was not disrupted. From these studies, it is evident that Golgi-targeting information is located primarily in the C-terminus of CoV E, but it appears that for some CoVs, like SARS-CoV E, additional targeting information could be found in the N-terminus.

#### Topology

A variety of different E protein topologies have been described and proposed for the different CoVs. Some studies have used prediction programs with conflicting predictions between the programs and some in conflict with the experimental evidence (Table 2). Infection and transient transfection experiments have shown that the C-terminus of the IBV E is located cytoplasmically while its N-terminus is located in the lumen of the Golgi complex [60]. The C-terminus of MHV E is also located cytoplasmically, but no N-terminus was detected. Based on the hydropathy plot of the protein, the authors suggested that it might be buried inside the lipid bilayer [71]. The C-terminus was confirmed to be in the cytoplasm and that the highly hydrophobic N-terminus causes it to be buried within the Golgi membrane [94]. Conversely, the TGEV E protein exhibits a topology of a luminal C-terminus and a cytoplasmic N-terminus [95]. To date, however, the topology of the SARS-CoV E protein has received the most attention. A FLAG-tagged SARS-CoV E protein, Yuan, Liao [91] was reported to assume an N- and C-terminus cytoplasmic topology. Prediction software demonstrated conflicting predictions between both the software and the experimental evidence; TMHMM and MEMSAT predicted a cytoplasmic N-terminus and a luminal C-terminus, while HMMTop predicted a luminal N-terminus and a cytoplasmic C-terminus. Moreover, transfected and infected cells expressing untagged SARS-CoV E exhibited a luminal N-terminus and a cytoplasmic C-terminus topology [66]. Given the variety of different topologies, the number of TMDs for the CoV E protein have also been inconclusive.

**Table 2:** Prediction programs showing membrane topologies of four different CoV E proteins with predicted locations of N- and C-termini, and TMDs. Prediction programs used: TM Pred, HMMTop, TMHMM 2.0, MEMSAT3, and MEMSAT-SVM [96–100]. Taken from Ruch and Machamer [41]

Prediction Program	IBV E			MHV E			SARS E			TGEV E		
	N	C	TMDs	N	C	TMDs	N	C	TMDs	N	C	TMDs
TM Pred	lumen	lumen	2	lumen	cyto	1	lumen	cyto	1	lumen	cyto	1
HMMTop	lumen	lumen	2	cyto	cyto	2	lumen	cyto	1	cyto	cyto	2
TMHMM 2.0	lumen	lumen	2	lumen	cyto	1	cyto	lumen	1	lumen	cyto	1
MEMSAT-SVM	lumen	lumen	2	lumen	lumen	2	lumen	lumen	2	cyto	lumen	1
MEMSAT3	cyto	cyto	2	lumen	cyto	1	lumen	lumen	2	lumen	cyto	1

The prediction programs in Table 2 likely conflict in their predicted outcomes based on the algorithm used by each program and/or the window size that was used to calculate the result. The design of algorithms used in prediction programs requires an array of aspects to be taken into consideration, largely those involved in machine learning, which makes identifying the exact reason(s) for the difference in predictions between programs challenging [101]. Nevertheless, the main reason likely stems from differences in the features unique to each algorithm, such as, whether the algorithm would include multiple features of the target protein(s) or only a clearly defined set of criteria; how accurately the algorithm should discriminate between the different features; the point at which the specificity or sensitivity for a certain feature is defined as too broad or too narrow [102]. The calculations used to design the algorithm along with its cut-off values should also be taken into consideration, all of which only speak to one aspect of machine learning. Nevertheless, some proteins prove challenging to isolate and not all biochemical techniques offer the needed high-resolution structural detail, in which case prediction programs are a good alternative and offer valuable insight into the predicted outcomes [101].

Many prediction programs also make use of a sliding window method to predict certain structural features of a protein. It is based on a window size that covers defined fragments of the amino acid sequence, rather than the whole sequence and takes into account that a given characteristic of an amino acid is not only determined by the residue itself, but also by the adjacent residues [103]. This method is widely used in the prediction of hydrophobicity, flexibility and rigidity, secondary structure, and tertiary structure of proteins [104–108]. It is possible that a standard window size, corresponding to a stretch of residues in the sequence, was not used between the prediction programs, or even between different CoVs, which might have resulted in the different topological predictions for each of the CoVs in Table 2. Based on a probabilistic approach, the prediction of structural features, such as coils and strands, would benefit from smaller window sizes as residues up to three and six positions away from the central residue, respectively, can affect the formation of these structures. Conversely, helical structure formation can be affected by up to nine residues away from the central residue and would benefit from a larger window size [103]. Accordingly, the use of a standardised, optimal window size could prove beneficial to obtain a more consistent and accurate topological prediction for CoV E.

The experimental evidence described in the previous section strongly suggests that the presence of an epitope tag does not interfere with the localisation of the CoV E protein. However, the use of epitope tags has been criticized for its interference with the properties or features of the tagged protein [41, 66]. By tagging the N-terminus of the IBV E protein with a FLAG tag, Ruch and Machamer [109] succeeded in producing a membrane hairpin conformation, with the N- and C-termini oriented cytoplasmically. However, the untagged E protein exhibited the topological conformation of a single transmembrane-spanning protein, demonstrating that the topology may be altered by the presence of the N-terminal tag [66]. Other reports proposing the hairpin conformation have also made use of N-terminal epitope-tagged CoV E proteins [91, 109].

The rationale for the multiple membrane topologies has been suggested in that, between the different CoV species, the E protein might not exhibit a uniform membrane topology or that the orientation of E varies depending on the level of protein expression or oligomerization [69]. Alternatively, the function of the E protein might dictate its membrane topology, depending on whether it is required to function as an ion channel or its involvement in the viral envelope during assembly [41].

### Post-translational modifications

#### Palmitoylation

Palmitoylation functions in the subcellular trafficking of proteins between membrane compartments and can also modulate protein-protein interactions (PPIs) [110, 111]. Palmitoylated proteins have an increased hydrophobicity, which has been reported to assist in membrane association and also functions in membrane anchoring [112, 113]. Palmitoylated viral proteins are well-represented in enveloped viruses, including the haemagglutinin (HA) protein of the influenza virus, Env of retroviruses and filoviruses, and F13 L of the vaccinia virus [114]. In the vaccinia virus, palmitoylation of its F13 L protein has been shown to be essential for targeting to the appropriate membranes [115]. The hepatitis C virus (HCV) nucleocapsid core protein binds to ER membranes in a palmitoylation-dependent manner for the formation of viral particles [116].

Of the CoV E proteins, only IBV, SARS-CoV, and MHV have been found to be palmitoylated [73, 93, 117]. A number of integral membrane proteins are substrates for palmitoylation where the cysteine residues adjacent to the TMDs serve as the targets [118, 119]. Double or triple mutation of the cysteine residues on the MHV-A59 E protein to alanine significantly reduces VLP formation [52, 117]. Furthermore, triple-mutated E proteins are unstable, prone to degradation, and significantly reduces the viral yield of the corresponding recombinant MHV, suggesting that palmitoylation of E plays an essential part in the viral assembly of MHV [117]. Palmitoylation of IBV E does not affect its localization to the Golgi region, as cysteine-mutated E proteins are indistinguishable from their palmitoylated counterparts [93]. Interestingly, mutation of certain hydrophobic residues in the TMD along with all three cysteine residues of SARS-CoV E protein disrupted targeting to the Golgi [73]. Although the authors did not demonstrate the localization pattern of the triple-mutated E protein on its own, the results suggest that palmitoylation alone of the SARS-CoV E protein does not affect its localization. Rather, it is possible that a loss of both the Golgi-targeting information in the TMD and the palmitoylated cysteine residues leads to the loss of localization as well as membrane its association [65]. Lopez, Riffle [117] suggested that palmitoylation of the E protein might affect how it interacts with the membrane. The position of the palmitoylated cysteine residues in relation to the hydrophobic TMD likely increases the region’s affinity for the membrane, serving to alter or stabilise association between the protein and the membrane.

#### Myristoylation

Linkage of myristic acid (C14:0) to the N-terminal of a glycine residue found on some viral, cellular, or bacterial proteins, is known as N-terminal myristoylation [120–123]. Several viral proteins are myristoylated including the poliovirus VP4 protein, simian immunodeficiency virus (SIV) Gag protein, human immunodeficiency virus (HIV) negative regulatory factor (Nef) protein, and the pre-S1 protein of the hepatitis B virus (HBV) [124–127]. All of these proteins contain the conserved sequence motif _1_MGxxxS/T, where ‘x’ can be any amino acid [80]. Coronavirus E proteins, along with other members of the *Nidovirales* order, reportedly have no myristoylation motif and it is suggested to be a feature unique only to the *Arteriviridae* family in the order of *Nidovirales* [80]. However, there appears to be no experimental evidence to support this.

#### Ubiquitination

Ubiquitination and its counterpart, deubiquitination, are well-characterised post-translational modifications with that serve to maintain homeostasis through the regulation of cellular protein levels and their functions [128]. Viruses can exploit this component of the host cell machinery, or even encode their own ubiquitinating/deubiquitinating enzymes to drive the viral life cycle [129]. Only SARS-CoV E has so far been reported to be ubiquitinated, although the relevance has not yet been determined. The SARS-CoV non-structural protein (nsp) 3 co-localises with E and its interaction was mediated through the N-terminal ubiquitin-like domain-1 of nsp3. Independently, a ubiquitination assay further demonstrated that E can be ubiquitinated and that its ubiquitination status inversely correlates to its stability and half-life [128, 130]. Moreover, given the late expression of SARS-CoV accessory protein 8b, Keng, Åkerström [130] suggested that it might function to modulate viral production by down-regulating E production and in doing so maintain an optimal viral titre. However, this will need to be confirmed in the context of a natural infection.

#### Glycosylation

In N-linked glycosylation, oligosaccharide moieties are attached to specific asparagine residues located in the consensus sequence Asn-X-Ser/Thr. It aids in the proper folding and trafficking of cellular and viral proteins by actively recruiting host chaperone proteins such as calnexin and calreticulin [131]. Very little information is available on the glycosylation of CoV E and its role. The IBV E protein has been suggested to contain a single glycosylation site in its luminal N-terminus, while SARS-CoV E has been predicted to contain two potential glycosylation sites [132]. Based on the topology of IBV E, Corse and Machamer [60] proposed that it could be glycosylated on asparagine residue five (N5) of the N-terminus. However, this was found not to be the case, likely due to the proximity of the residue to the membrane [133]. Likewise, residue N48 in SARS-CoV E was also shown not to be glycosylated and proposed to be non-functional for the same reason [73]. Conversely, residue N66 was shown to be glycosylated and, more interestingly, mutation of this residue generated higher molecular weight forms resembling dimers and trimers of the E protein. This suggests that glycosylation of N66 might function to prevent oligomerization of the E protein, possibly to promote a specific role of the E protein. Accordingly, multimeric forms of the E protein may not be glycosylated on N66 possibly to promote the functioning of E in other capacities [134]. Westerbeck and Machamer [90] used both infected and transfected cells and reported the presence of two different forms of the IBV E protein, each associated with a specific function. They proposed that the lower molecular weight, possibly monomeric form, functions in disruption of the host secretory pathway, while the higher molecular weight oligomeric form is required for virion assembly. Clearly, more research is needed to determine whether all CoV E proteins are glycosylated, or whether it is unique to SARS-CoV that might confer to it certain pathogenic features, and what the importance of E protein glycosylation is.

### Protein-protein interactions: Intraviral

#### Membrane and envelope proteins

Co-localization of and interaction between M and E is probably the most well-established and characterised of PPIs between the CoV structural proteins [56, 60, 61, 117]. Co-expression of M and E is sufficient for VLP formation and release [37, 60–64]. The interaction is mediated by the C-termini of both proteins and occurs on the cytoplasmic side of the ERGIC [56, 61, 89]. The importance of these domains is evident by the drastic reduction of VLPs upon deletion of the domains [56].

#### Envelope and envelope proteins

The CoV E protein is unique in that it can form homotypic interactions, which allows it to oligomerise and generate an ion-channel protein known as a viroporin [135, 136]. Biochemical evidence suggests that the ability of CoV E to form homo-oligomeric multimers is dependent on its TMD. Synthetic peptides that correspond to the SARS-CoV E TMD can form dimers, trimers, and pentamers, demonstrating the importance of the TMD in CoV E homotypic interactions [137]. This was ability to produce multimeric homo-oligomers was confirmed by expression of SARS-CoV E in Sf9 insect cells. Substituting certain hydrophobic residues in the TMD with charged residues, significantly alters the electrophoretic migration rate of E to the extent that only monomers are observed [73]. To date, not many studies have investigated which TMD residues are required for CoV E homotypic interactions. Mutation of the TMD residues asparagine 15 (N15) to alanine (N15A) and valine 25 (V25) to phenylalanine (V25F) have been found to abolish the ion channelling capability of CoV E viroporin, a structure dependent on its homopentameric conformation [75, 76, 138]. Interestingly, mutation of N15A and V25F, respectively, appear to hamper the oligomerisation of CoV E, at least to some extent. The appearance of monomers in response to V25F clearly suggests that this residues plays a more crucial role in oligomerisation, as opposed to N15A, which appears to reduce the amount of pentamers only slightly [139]. The ability of CoV E to assemble into homopentameric structures is clearly important in the formation of a functional CoV E viroporin [75, 76, 135–138, 140].

#### Nucleocapsid and envelope proteins

It has been shown that M and E are sufficient to drive VLP formation in many CoVs and that its production is further enhanced by the co-expression of N [42, 60, 63, 64, 141]. It is thought that E and N interact with M independently and are assembled separately into VLPs. Accordingly, it is not known whether E and N interact and, in doing so, if this interaction is what could enhance virion production. Only two studies have reported a possible interaction between E and N, one for murine MHV and the other for SARS-CoV. Tseng, Wang [142] reported an interaction between SARS-CoV E and N mediated largely through the C-terminus of both proteins. Deletion of the last C-terminal residue of E markedly reduced E and N interaction although it did not seem to significantly compromise efficient VLP production. Although the study only looked at an E-N interaction in transfected cells, it is interesting to note that Maeda, Maeda [143] already found coimmunoprecipitation of structural proteins E and N in MHV-infected cells. This suggests that there might, in fact, be a physical interaction between E and N but the reason and exact requirements for this interaction remains to be determined. More research is needed to understand this interaction and whether it offers a possible explanation as to why or how VLP production is enhanced during the co-expression of M, E, and N [42, 52].

#### Spike and envelope proteins

**Fig. 2:**
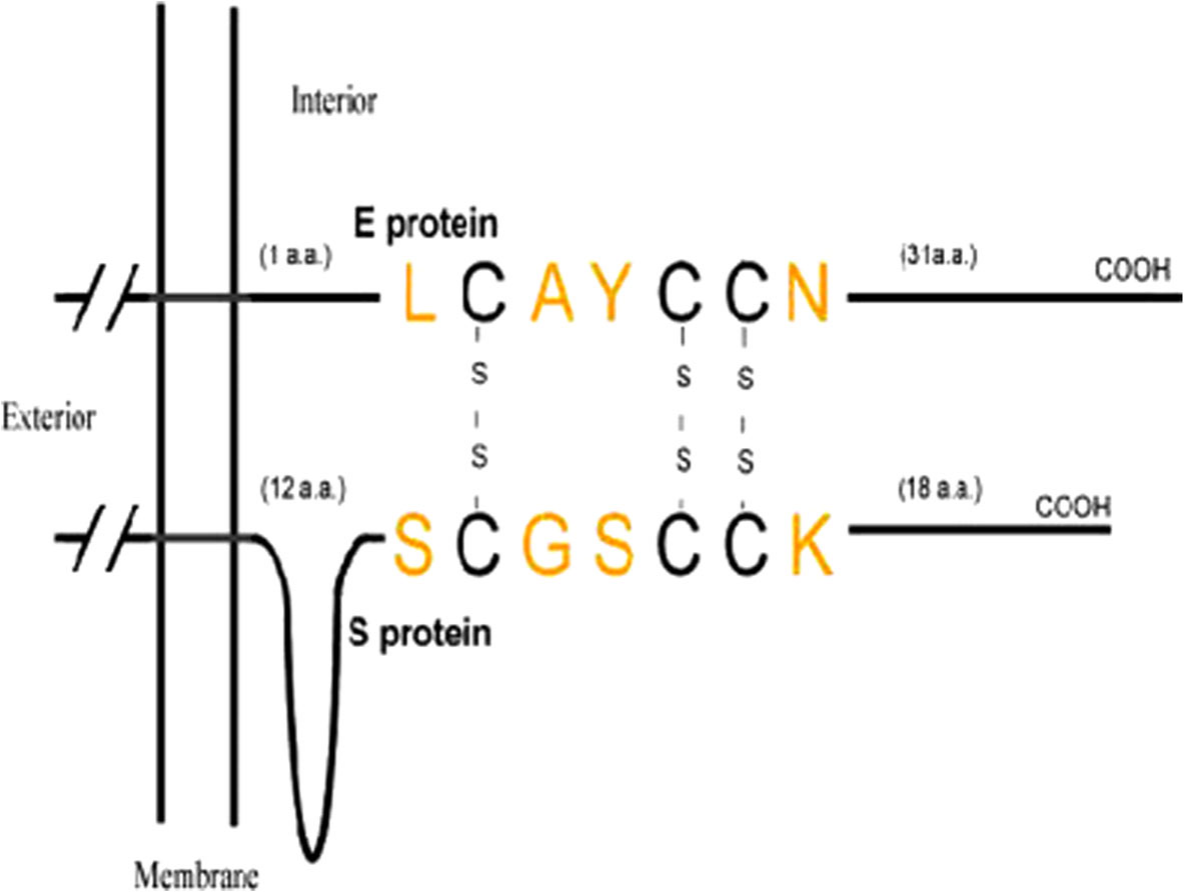
Predicted interaction between SARS-CoV E and S proteins through disulphide bonds [79]

A sub-regional analysis of both E and S revealed a triple cysteine motif located directly after the E protein TMD (NH_2_- … L-**Cys**-A-Y-**Cys**-**Cys**-N … -COOH) and a similar motif located in the C-terminus of S (NH_2_- … S-**Cys**-G-S-**Cys**-**Cys**-K … -COOH) [79]. The authors proposed that the predicted orientation, position, and composition of these two motifs could serve as a structural basis for the association between E and S, which would be mediated by the formation of disulphide bonds between the corresponding cysteine residues (Fig. 2). Although this is yet to be proven experimentally, it would be interesting to see whether this interaction is indeed possible. Such evidence could also provide some insight into the debated topological conformations of the E protein and could confirm whether multiple topologies are possible to accommodate this interaction.

Experimental data on a physical interaction between CoV S and E is extremely limited with the exception of one study, which showed that SARS-CoV S is an interacting partner of E [128]. Using a tagged E protein, the study aimed to identify SARS-CoV E protein interacting partners by a tandem affinity purification (TAP) system coupled with mass spectrometry (MS; TAP-MS). Although S was shown to co-purify with E, the authors did not pursue the mechanism or importance of this interaction. This finding clearly warrants further investigation into an intraviral protein interaction which has not been investigated yet.

Protein 7a, a structural protein unique to SARS-CoV, is incorporated into mature virions and plays an important part in the pathogenesis of SARS-CoV, where it functions to induce apoptosis, arrest the cell cycle, and promote the production of pro-inflammatory cytokines [144–148]. In a mammalian two hybrid system, SARS-CoV E was found to interact with 7a, but the importance of this interaction has not yet been determined [149]. However, despite this interaction with E, 7a still appears to be dispensable for SARS-CoV replication both in vivo and in vitro [30, 150–152].

### Protein-protein interactions: Host-viral

Viruses lack the necessary machinery to self-replicate and are, therefore, dependent on the host cell machinery for propagation. Numerous viruses exploit the host cell’s replication machinery to establish an infection by way of host-viral PPIs [83]. The anti-apoptotic protein B-cell lymphoma-extra-large (Bcl-xL) protein was the first host protein reported to interact with SARS-CoV E protein, alluding to the possibility that the coronaviral E protein is also capable of host-viral PPI [87]. The domain mediating this PPI was only identified later when the SARS-CoV E protein was shown to interact with the protein associated with *Caenorhabditis elegans* lin-7 protein 1 (PALS1) [82]. It is now established that PALS1 bound to SARS-CoV E through its PDZ domain. The PDZ domain is a protein-protein recognition sequence found in cellular adaptor proteins that coordinate host cell signalling pathways by binding to other proteins that have a complementary PBM. A number of these signalling pathways and processes are exploited by viruses for replication, propagation, and pathogenesis [153–157]. The PBM of SARS-CoV E is found in the last four amino acids (DLLV) of its C-terminus [1, 82].

To date, E has only been reported to interact with five host proteins, i.e. Bcl-xL, PALS1, syntenin, sodium/potassium (Na^+^/K^+^) ATPase α-1 subunit, and stomatin [18, 66, 82, 87]. Some context has been offered as to the relevance of each interaction, but it is not yet fully understood. Yang, Xiong [87] proposed that the interaction between E and Bcl-xL contributed to the SARS-CoV-induced lymphopenia observed in most SARS patients. Teoh, Siu [82] reported that the E–PALS1 interaction disrupts tight junctions in the lungs, suggesting a mechanism whereby SARS-CoV virions can breach the alveolar wall and develop into a systemic infection. Nieto-Torres, DeDiego [66] suggested that the interaction of E with Na^+^/K^+^ ATPase α-1 subunit and stomatin, 2 proteins involved in maintaining ionic homeostasis, could be responsible for the reduced levels and activity of human epithelial sodium channels. Jimenez-Guardeño, Nieto-Torres [18] is the only group to have shown that E is a determinant of SARS-CoV pathogenesis in vivo. By infecting mice with recombinant SARS-CoV viruses, they demonstrated that E caused syntenin to be redistributed to the cytoplasm where it triggered an overexpression of inflammatory cytokines. This would give rise to an exacerbated immune response, resulting in tissue damage, oedema, and culminate in the characteristic acute respiratory distress syndrome (ARDS).

**Fig. 3:**
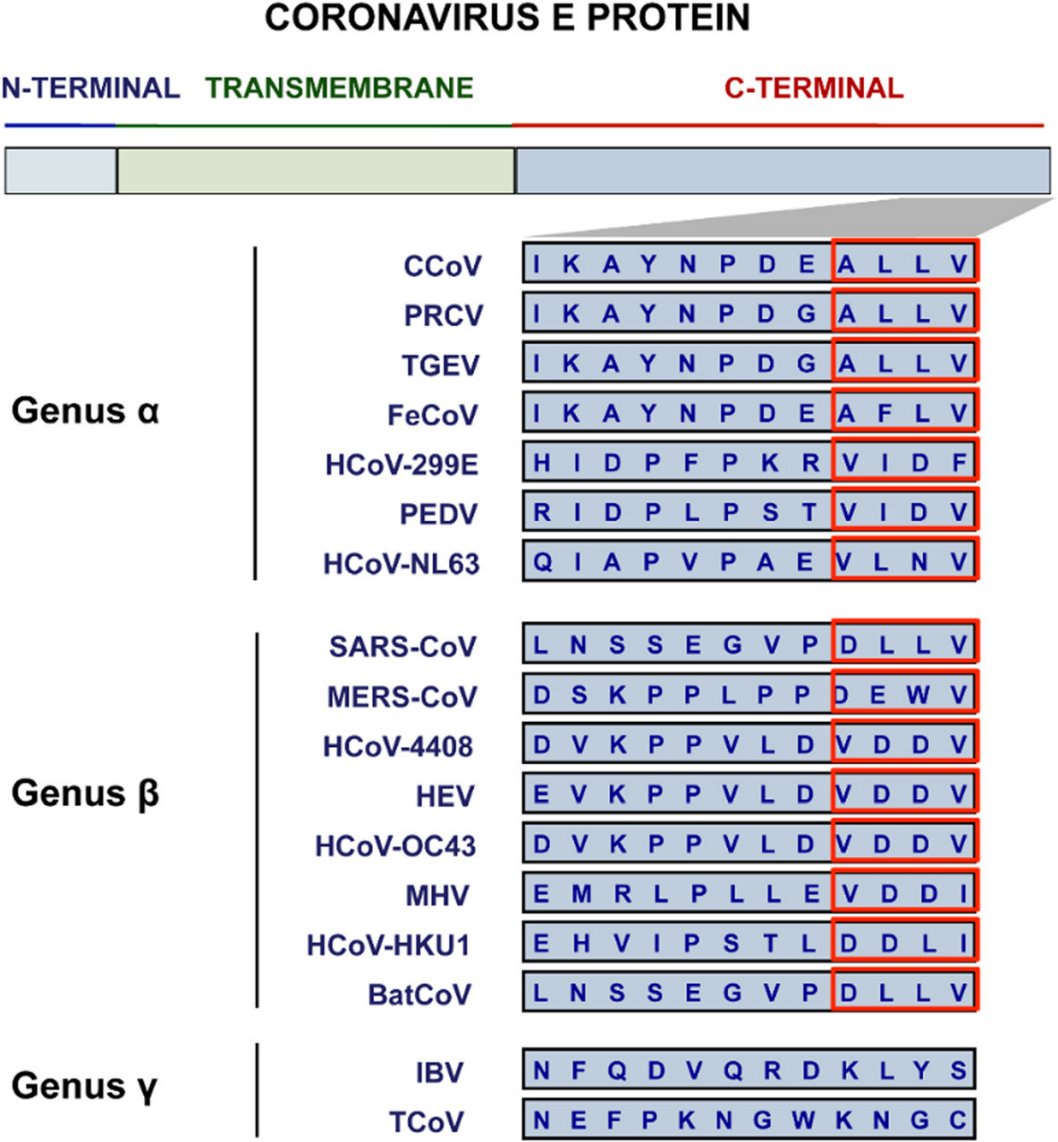
Partial amino acid sequences of the E protein C-terminus for the different CoV genera. Red blocks represent the potential location of the predicted PBM motif [18]

Interestingly, each of the E protein interactions was only reported in SARS-CoV. A closer look at the predicted PBM motif for each of the coronaviral genera α, β, and γ reveals that the PBM motif appears to be conserved only among the α and β CoVs and is not found in the γ CoVs (Fig. 3) [18]. As no experimental evidence yet speaks to any such interactions for the other α and β CoVs, it remains to be seen whether the reported interaction partners uniquely interact with SARS-CoV E, or if they can also interact with E from other coronaviral species from the same genus. Aside from this, it is of therapeutic importance that more E interaction partners be identified as inhibitors of p38 mitogen-activated protein kinase (MAPK) were shown to increase the survival rate of mice, protecting them from a lethal infection [18, 158]. Identifying more interaction partners for CoV E could provide a more targeted therapeutic approach where licensed coronaviral treatments are currently ineffective [26–28].

### Functions of the envelope protein

Despite its enigmatic nature, research conducted to date has been able to propose three roles for the CoV E protein. The interaction between the cytoplasmic tails of the M and E proteins drives VLP production, suggesting that E participates in (1) viral assembly [56, 61, 89]. The hydrophobic TMD of E is also crucial to the (2) release of virions [40, 53, 159]. Lastly, SARS-CoV E is implicated in the (3) pathogenesis of the virus [18, 82, 87]. The progress made in these three aspects of E will be reviewed accordingly.

#### Assembly and budding: Membrane curvature

Coronaviruses are unique among enveloped viruses in that assembly of the viral envelope occurs at the ERGIC. From there, virions bud into the lumen, navigate their way through the host secretory pathway, and ultimately egress from the cell [89, 90, 160, 161]. Although assembly of the viral envelope is coordinated by M, both M and E are required for the production and release of VLPs [51, 55, 56, 60–64, 141, 162–164]. Still, deleting the E gene from several recombinant CoVs does not halt virus production but rather cripples viral production severely or produces replication-competent but propagation-defective virions [35, 39, 40, 67, 68, 150, 165, 166]. Clearly then E is involved in the CoV assembly and release, but the exact role is not yet fully understood.

The coronaviral envelope consists predominantly of M while only a small portion of E is incorporated into the viral envelope of virions [100, 167, 168]. Extensive electron microscopy (EM) studies conducted on M from a variety of CoVs provided no indication that M is capable of inducing membrane curvature on its own [51, 169, 170]. In fact, various recombinant CoVs (rCoVs) lacking the E gene (ΔE) exhibit a strikingly aberrant morphology. When C-terminus residues of MHV E were mutated to alanine, virions became temperature sensitive and took on pinched, elongated shapes rather than the typical spherical particles observed among wild type virions [171]. Plaques of recombinant MHV-ΔE exhibited a very similar aberrant morphology, presenting as small, irregular-shaped plaques with jagged edges [39]. Cells infected with recombinant SARS-CoV-ΔE (rSARS-CoV-ΔE) contained a lower number of mature virions but exhibited a higher proportion of vesicles containing a dense, granular material. This material was proposed to be the result of the aborted viral assembly process that gave rise to immature virions [35]. Most interestingly, TGEV-ΔE-infected cells contained immature virions that were blocked from being secreted into the medium. The absence of TGEV E arrested virus trafficking and, thereby, blocking full virion maturation [40]. In comparison, the phenotype of VLPs made up of M and E are described as smooth and indistinguishable from, or resembling, wild type virions, placing this morphology in stark contrast to that observed of virions lacking E [37, 63, 64]. Clearly, even though viral assembly and production is not completely stopped in the absence of E, the aberrant morphology of ΔE-virions strongly suggests that E participates in the assembly process. Most likely then, instead of coordinating viral assembly, the function of E is rather to induce membrane curvature of the viral envelope, thereby allowing CoV particles to acquire their characteristic spherical shape and morphology.

**Fig. 4:**
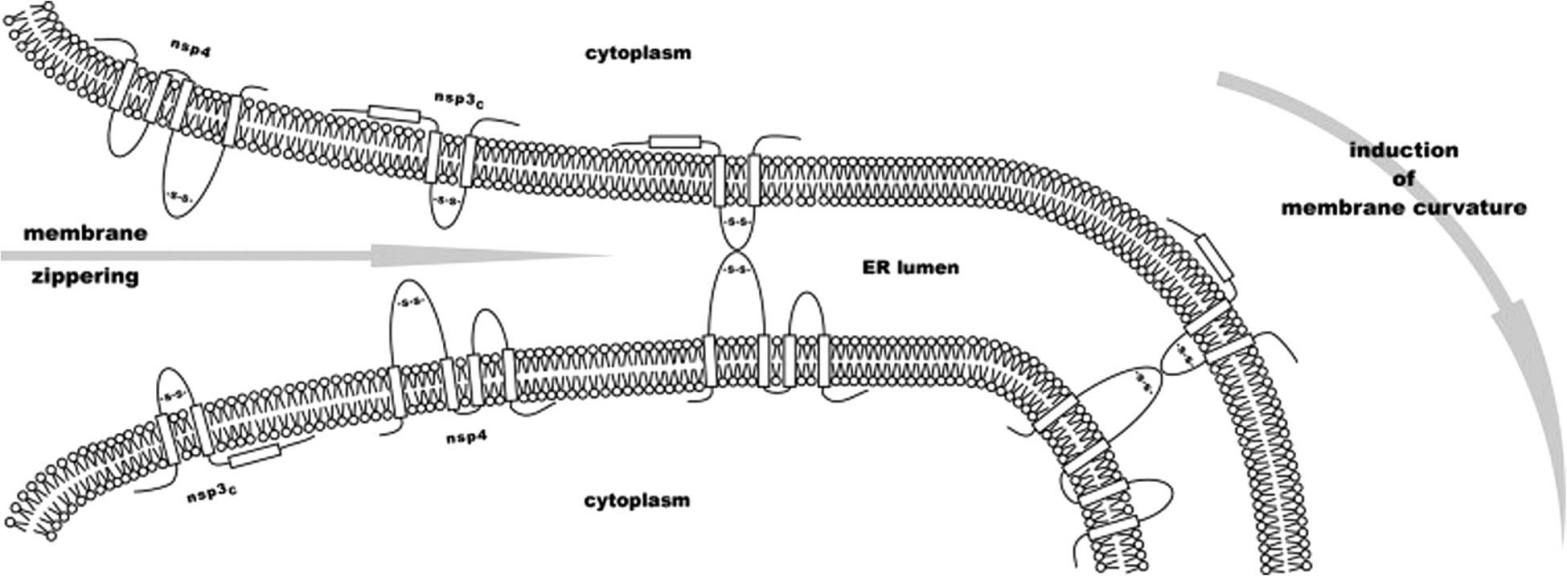
Model proposed by Hagemeijer, Monastyrska [177] for the induction of ER membrane curvature. The luminal loops of CoV nsp3 and 4 are required to initiate rearrangement of the ER membrane and produce the DMVs characteristically seen in CoV-infected cells

Coronavirus-infected cells contain several different membranous structures, including double-membrane vesicles (DMVs) and convoluted membranes (CMs) [172–175]. However, little is known about exactly how these structures are formed and which viral and/or host proteins are involved in this process. Co-expression of SARS-CoV nsps 3, 4, and 6 can induce membrane rearrangements that resemble the DMVs and CMs observed in CoV-infected cells [176]. The luminal loops present in full-length nsp3 and nsp4 are essential for the formation of the replicative structures seen in SARS-CoV-infected cells [176, 177]. Moreover, the cysteine residues located in the luminal loop nsp4 appear to be particularly important in the process of ER membrane rearrangement [177]. Hagemeijer, Monastyrska [177] proposed a model in which the luminal loops located between the transmembrane regions of nsp3 and 4 interact with one another to initiate the rearrangement of ER membranes and induce membrane curvature to form DMVs (Fig. 4).

This underpins the importance of establishing a unanimous topology for the E protein as this model could be applied to the induction of membrane curvature by E, provided E can assume multiple topologies during an infection. Should it be demonstrated that E can take on a topology with a luminal loop, this would not be inconceivable as a possible mechanism for the induction of membrane curvature initiated by E or in which E participates. Equally, as heterotypic interactions of nsp3 and 4 are required to induce ER membrane curvature, and the expression of both M and E is required for the formation of smooth, spherical CoV VLPs, it would be interesting to see if a heterotypic interaction between M and E could drive membrane curvature by a similar mechanism [176–178]. Alternatively, no research exists on the exact purpose of the N-terminus of E. Perhaps homotypic interactions mediated by the N-termini of alternating E proteins could be responsible for inducing membrane curvature by a similar mechanism. It is also worth noting that the mutation of each of the cysteine residues located in the nsp4 luminal loop abrogated the ability of nsp4 to rearrange the ER membranes [177]. This is interesting because cysteine residues are substrates for the palmitoylation of proteins associated with membranes [113]. Perhaps this corroborates the requirement of E palmitoylation, not in assembly per se, but rather by anchoring E during the induction of membrane curvature. It is quite evident that although a lot of progress has been made in determining the role of E in assembly, much still remains unknown. The role of E has also been proposed to be merely catalytic by functioning to pinch off, or in the scission of, the viral particle from the ER membrane during the terminal phase of budding [63].

#### Assembly and budding: Scission

The viral envelope is formed primarily during assembly and culminates when the virion buds from the host membrane, a process known as scission [179]. Broadly, enveloped viruses can accomplish membrane scission either by hijacking/exploiting the host cell’s scission machinery or through the expression of their own scission proteins [179]. In the absence of scission machinery, the budding process begins but ultimately stops, and render budding virions attached to the membrane by a small membranous neck. This causes virions to have an uncharacteristically elongated morphology sometimes referred to as “beads-on-a-string” and is seen in viruses that lack the necessary machinery to release the budded virion [179–183]. This is clearly and elegantly demonstrated in the mutation of the matrix-2 (M2) protein, a viral protein responsible for the budding and scission of the influenza virus. Virions that have failed to undergo scission remain attached to the host cell membrane by a membranous neck. The budding process is reinitiated at the site where scission failed, and a new virion is formed. However, the new virion also remains attached to the membrane as well as the previous virion by a small membranous neck. The continuation of this cycle and repeated initiation of budding results in the formation of consecutive scission-defective virions that resemble beads on a string [180, 181]. The same morphology has been reported for the Moloney murine leukaemia virus upon deletion and mutation of p12 protein that functions in its assembly and release [182].

While some enveloped viruses, like influenza A virus, encode their own scission proteins, other viruses rely on the host cell’s endosomal sorting complex required for transport (ESCRT) to accomplish this [179]. This demonstrates a necessity for viral-host PPIs but given the shortage of information available on CoV E-host PPIs, it is nearly impossible to say whether E mediates scission in an ESCRT-dependent manner or not. It is, therefore, essential that host cell candidates capable of interacting with CoV E be identified as they could be potential therapeutic targets for CoV antivirals to stop CoV scission. Conversely, the release of influenza virions is mediated by the M2 protein in an ESCRT-independent manner. The amphipathic helix located in the cytoplasmic tail of the M2 protein is both required and sufficient for the detachment of vesicle buds in an in vitro model system [184]. Mutation of the hydrophobic region of the helix also significantly reduced viral release in vivo, confirming the importance of the 17-amino-acid-helix in the release of the influenza virus in vivo as well. In the absence of the M2 protein, buds formed inside infected cells but failed to detach and such cells exhibited the beads-on-a-string morphology. This suggests that M2 can serve as a substitute for ESCRT complexes during influenza virus budding and, more importantly, raises the possibility of functionally equivalent M2’s in other enveloped viruses.

Interestingly, an amphipathic α-helix is predicted to be located in the TMD of CoV E and has even been confirmed in some of the CoVs [72, 76, 77, 135, 136, 138, 140, 159, 185, 186]. It appears that no attempts have been made to determine whether E of any of the CoVs is responsible for the scission of CoV virions during budding. However, expression of E alone has been reported to produce and secrete vesicles from cells but no further research has been done to determine how this is possible [60, 143]. Mutational studies would certainly benefit from EM analysis to determine what effects TMD mutations of E would have on virion budding. Electron microscopy can clearly demonstrate the consequences of mutated scission proteins and can even prove useful to ascertain what effects complete gene deletion have on viral budding.

#### Release: Viroporin

**Fig. 5:**
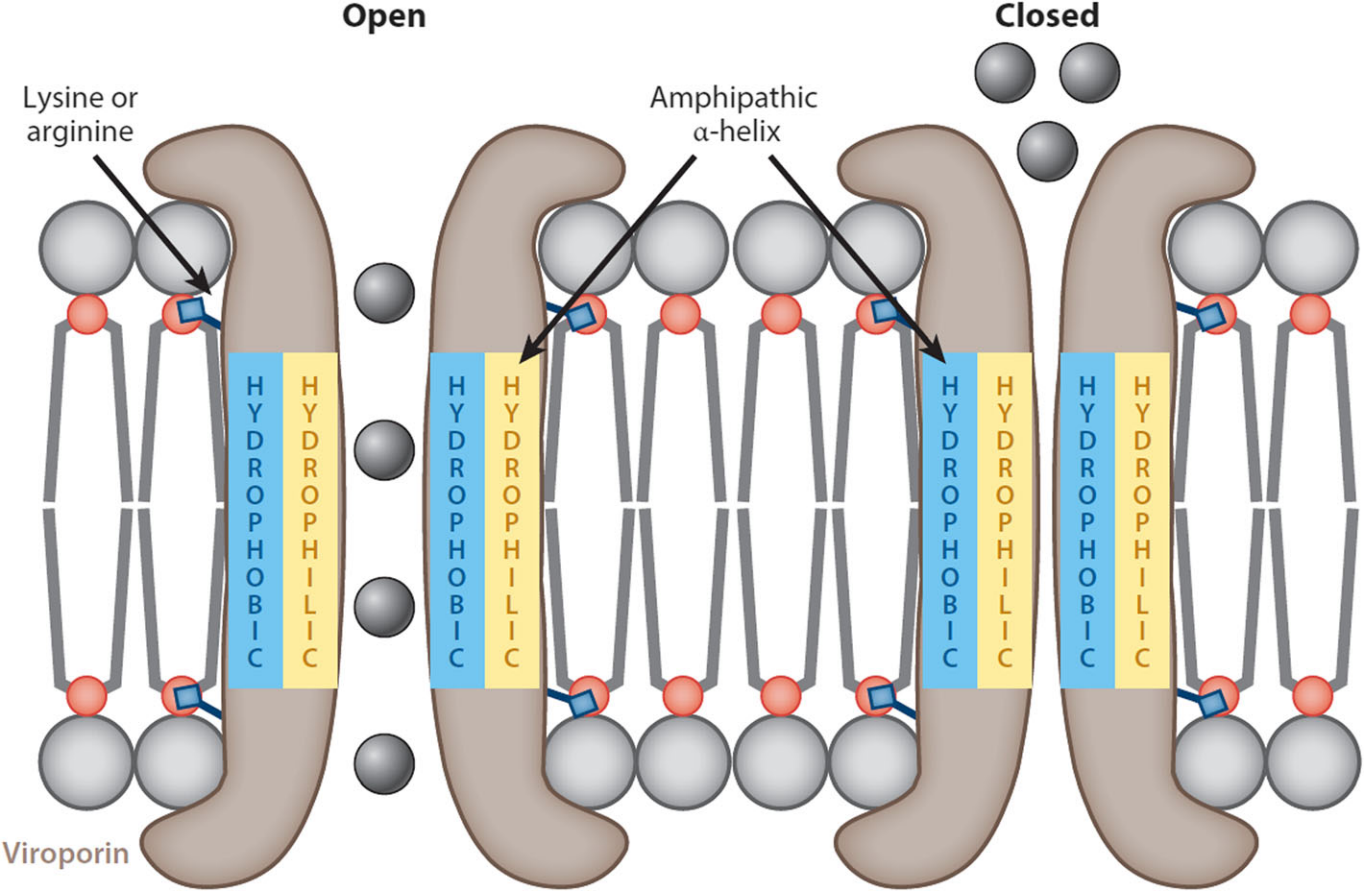
Illustration of a typical viroporin structure and motifs. The pore of the viroporin (brown) is created by the amphipathic α-helix and the viroporin is anchored to a lipid bilayer by terminal positively charged residues (lysine or arginine). Conformational changes in the structure regulate the flow ions through the viroporin by opening (left) and closing (right) the pore [208]

While the accumulation of E at the ERGIC points largely to a role in assembly and budding, only a small portion is incorporated into the viral envelope, suggesting that E has additional functions centred around the ER and Golgi region [66, 92, 109, 159]. Viroporins are viral-encoded membrane pore-forming proteins that can modulate cellular ion channels and have been suggested to regulate and function in multiple stages of the viral life cycle, from viral entry to assembly and release, and even pathogenesis [184, 187–196]. Although viroporins are not essential to viral replication, their absence does weaken or attenuate the virus and diminishes its pathogenic effects [35, 197–200]. They tend to be small proteins (~ 60–120 amino acids) of a predominantly hydrophobic nature that oligomerise in the membranes of infected cells, forming hydrophilic pores. The hydrophobic residues line the outside of the structure, oriented toward the phospholipids, while the inside of the pore is made up of the hydrophilic resides [140, 159, 201–204]. Most viroporins share certain structural features such as an amphipathic α-helix in the hydrophobic domain (HD) along with a cluster of positively charged, basic amino acids (such as lysine or arginine) which anchor the pore to the membrane through electrostatic interactions with the negatively charged phospholipids (Fig. 5) [187, 205–207].

Viroporins can transport different ions but appear to be largely selective for the positively charged ions hydrogen (H^+^), K^+^, Na^+^, and calcium (Ca^2+^) [209, 210]. Although preferentially selective for cations, viroporins can also transport anions. The preference simply appears to be for cations over anions [211–213]. It is, however, interesting to note that, at a neutral pH, the ion selectivity of the respiratory syncytial virus (RSV) small hydrophobic (SH) protein can change from cationic to anionic [214]. This suggests that viroporins are sensitive to changes in the cellular environment, a property that could be of therapeutic value. After all, the influenza A virus M2 protein is pH-gated and activates upon acidification of the endosome following receptor-mediated endocytosis of the virus [215]. In the same study, Schnell and Chou [215] showed that the anti-viral drug rimantadine exerts its anti-viral property by stabilising the M2 viroporin in its closed conformation and in doing so inhibits viral replication [209, 216]. Similarly, the E protein of several CoVs possesses ion channel activity, though the only structural data of the CoV viroporin has been derived from SARS-CoV using synthetic peptides [75, 135, 136, 138, 217, 218].

Synthetic peptides of SARS-CoV E demonstrate that the TMD is responsible for its ion-conductive properties [135, 136, 138]. Computational predictions and spectroscopic studies show that the SARS-CoV E TMD undergoes oligomerisation, characteristic of ion-channelling proteins, to form a stable pentamer [75, 135–137]. Viroporin formation appears to be mediated by ionic interactions rather than disulphide bonds as mutation of the porcine reproductive and respiratory syndrome virus (PRRSV) E protein cysteine residues appears to be dispensable for oligomerisation [219]. Research into the mechanism of viroporin formation is hampered by the hydrophobic nature of the TMD and has thus far been limited largely to mutational studies and the use of ion channel inhibitors such as amantadine and hexamethylene amiloride.

The CoV E viroporin is equally cation-selective when it comes to its ion-channelling properties, demonstrating a preference for the monovalent cations Na^+^ and K^+^ [217, 218]. Synthetic peptides of SARS-CoV E, that resemble the CoV E viroporin, are able to transport Na^+^, K^+^, and chloride ions (Cl^−^) but are more selective of Na^+^ over K^+^ and least selective of Cl^−^ [217]. Synthetic peptides that correspond to E from HCoV-229E, MHV, and IBV exhibit a similar cation-selectivity for MHV and IBV E as for SARS-CoV E. However, it is interesting that although the E viroporin synthetic peptides of HCoV-229E were still cation-selective, it exhibits a slightly higher selectivity for K^+^ than for Na^+^ [218]. The SARS-CoV E synthetic peptide findings were corroborated using a full-length SARS-CoV E protein [76]. More recently, purified full-length MERS-CoV E has also demonstrated limited ion-channelling properties and would benefit from a more comprehensive characterisation to establish whether it has ion-channelling properties similar to that of the other CoVs [140].

It should be cautioned that the charge on the lipid head group of membranes used can modulate the ion-selectivity of the viroporin. Neutral lipids appear to negate the selectivity of the viroporin as the channels formed did not seem to differentiate cations from anions. In contrast, negatively charged lipids were more cation-selective than neutral lipids, being more permeable to cations [76]. This suggests that the lipid head group of the membranes in use should be taken into consideration when interpreting the results as it might skew the results and inaccurate conclusions may be drawn. At times, the ion channels were only marginally more selective of cations, bringing into question the ion-selectivity of the CoV E viroporin for one cation over another. In fact, an ion channel is only considered ion-specific when its permeability is nearly exclusive to one ion while extremely low to others [220]. Synthetic peptides corresponding to the full-length SARS-CoV E viroporin have also recently been shown to be capable of transporting Ca^2+^ and was linked to the inflammatory response often observed in ARDS [221]. This is the only study so far to have shown that the E viroporin of any CoV is capable of Ca^2+^ transport.

Recent efforts have been directed toward understanding how mutant CoV E viruses carrying ion channel-inactivating mutations revert to their original pathogenic state. Mutants of SARS-CoV E carrying mutations N15A and V25F in the TMD restored ion channel activity by incorporating compensatory mutations in both in vitro and in vivo systems [77]. Mutant N15A reverted by incorporating a single mutation that led to an amino acid change at the same position (A15D), creating a more stable mutant. Conversely, mutant V25F reverted to mutants with amino acid substitutions at either the same position (F25D) or positions relatively close to the original mutation (L19A, F20 L, F26 L, L27S, T30I, L37R). Intriguingly, the V25F mutants appeared as early as 2 days after mice were infected where revertant mutant T30I surpassed the growth of the original virus by day two. This suggests that while some of these mutations appear to merely restore the loss of ion channel activity, it is not entirely inconceivable that revertant viruses would acquire gain of function mutations that can render it more virulent [77]. Similar results were recently reported for IBV E TMD residues analogous to N15A and V25F (T16A and A26F) [222]. It is interesting to note that in both cases SARS-CoV E and IBV E followed a similar trend in their reversion: mutations at N15A and T16A both reverted by substitution of a single residue, whereas mutations at V25F and A26F produced revertants by acquisition of multiple residues.

Some viroporins have been implicated in the release of viruses, but it is not yet known whether the release is mediated by the ion channel activity of the proteins [187, 223–226]. An intriguing study recently reported that both IBV infected and IBV E transfected cells exhibited a marked increase in the pH of the Golgi lumen [227]. These findings suggest that the IBV E viroporin could channel H^+^ and possibly mediate viral release by its ion channel activity. However, this increase in pH was found only in cells expressing a monomeric form of IBV E and not the oligomeric form as required for viroporin formation. The authors proposed that the change in pH could be attributed to an interaction between the monomeric form of E and a host protein. Although possible, only a very small number of host proteins have been shown to interact with CoV E. The monomeric and oligomeric forms were produced by transfection of mutated IBV E A26 to F26 (E^A26F^) and T16 to A16 (E^T16A^), respectively. In an earlier study, the same authors demonstrated that these two forms were present in IBV E-infected cells but that the monomeric form was much less (~ 10%) in infected cells than in transfected cells (~ 50%). The oligomeric form, however, was the dominant form in infected cells [90]. This suggests that other viral proteins might affect or modulate the oligomerisation of IBV E. It is interesting to note that the M2 protein amphipathic helix motif was required for release of influenza A virus (IAV) particles, perhaps indicating that this motif might be required for the processes budding, scission, and for viroporin activity [181]. It might be worth investigating whether ion-channel inhibitors, such as amantadine, or proton pump inhibitors specifically are able to inhibit this increase in Golgi pH. For now, though, it still remains to be seen whether CoV release is mediated by viroporin ion channel activity or through PPIs with host proteins of the secretory pathway.

#### Pathogenesis: ER stress response/unfolded-protein response (UPR) and apoptosis

The ER can sustain a high load of protein content without being overwhelmed [228]. However, when the ER’s capacity for folding and processing proteins is exceeded, unfolded or misfolded proteins rapidly accumulate in the lumen and the ER stress response, or unfolded-protein response (UPR), is activated. The various signalling pathways that make up the UPR collectively function by enhancing the folding of proteins, chaperoning, and ER-assisted degradation (ERAD) [229]. If, however, the UPR is prolonged and irreversible, apoptosis will be initiated [230]. By increasing the protein content, folding, and processing of the ER, viral infections can also trigger the UPR and this pathway can be used by the host cell as an antiviral response [231]. Very few studies have looked at the role of CoV E in the ER stress response and its ability to induce apoptosis. In cultured cell lines, overexpressed MHV E and epitope-tagged SARS-CoV E induces apoptosis [87, 232]. However, cells infected with rSARS-CoV and rSARS-CoVΔE, a more biologically relevant system, demonstrated that SARS-CoV E may regulate the UPR as part of its pathogenesis [233]. Cells infected with SARS-CoVΔE exhibit a stronger stress response compared to cells infected with the wild-type virus. Moreover, a higher degree of apoptosis was observed in SARS-CoVΔE-infected cells than in those infected with the wild-type virus.

This study demonstrates the risk of interpreting data from overexpression and epitope-tagged studies. Results generated by such studies might offer some insight into the putative functions of viral proteins but should be interpreted with great care as they can be misleading. Findings can only be more conclusive when supported by results from studies in more biologically relevant systems. The study also shows that CoV E has an anti-apoptotic function in infected cells by suppressing the UPR during infection, likely as a survival mechanism and to continue viral propagation. This function of E has only been demonstrated in SARS-CoV so far, one of the most virulent HCoVs. It would be interesting to see whether E of the other CoVs, as well as the less virulent HCoVs, are also able to contribute to pathogenesis by regulating the host cell stress response.

#### Immune response: Inflammasome activation

Viruses often encode proteins that interfere with the immune system to either inhibit a response or enhance one as part of their pathogenicity. Some viral proteins disrupt components of the immune response pathways to disrupt the immune system and promote their viral evasion and pathogenesis [234–237]. Alternatively, viral proteins can modulate other cellular factors that could also disrupt the immune response to promote pathogenesis. Coxsackievirus 2B protein promotes the internalisation of major histocompatibility complex class I (MHC-I) proteins and, in doing so, prevents their transport to the cell surface for immune recognition [238]. This protein also delays the transport of proteins along the secretory pathway by altering the Ca^2+^ and H^+^ concentrations of the Golgi and ER compartments and has been proposed to be a mechanism of immune evasion as well [239]. Influenza virus M2 protein triggers activation of the NOD-like receptor family, pyrin domain containing 3 (NLRP3) inflammasome by creating ionic imbalances through its ion-channel activity [240]. Other viruses use viroporins to stimulate an immune response as part of their pathogenicity, including the E protein of PRRSV [241–243].

Inflammasome activation by CoV E was first reported in PRRSV [242]. Blocking ion channel activity with amantadine significantly inhibited activation of the inflammasome, demonstrating an association between E viroporin activity and inflammation. Recently, the transport of Ca^2+^ by SARS-CoV E was shown to trigger inflammasome activation [221]. This establishes the link between inflammasome induction by SARS-CoV E and the inflammatory-mediated lung damage seen in SARS-CoV-infected mice [77]. Interestingly, despite attempts to inhibit ion channel activity in SARS-CoV E, by mutating N15A and V25F, viruses restored ion channel activity by incorporating additional mutations after several passages. The authors concluded that this ion-channelling function confers a selective advantage to the virus [77]. The reduction of inflammatory cytokines in the absence of CoV E ion channel activity suggests that inhibition of the CoV E viroporin limits CoV pathogenicity and could be of therapeutic value to CoV infections.

### Future perspectives and conclusion

While most CoV infections, such as those caused by HCoV-229E, HCoV-OC43, HCoV-NL63, and HCoV-HKU1, are mild and self-limiting, SARS-CoV and MERS-CoV cause severe infections that lead to high mortality rates [244–246]. There are currently no effective, licensed therapies for HCoV infections and existing treatment strategies are generally limited to symptomatic treatment and supportive care [26–28, 247]. While an extensive amount of research has gone into identifying potential treatment options, most have only shown promise in vitro and will likely not progress further as they often have one or more limitations. Anti-viral candidates either exhibit only a narrow spectrum of activity, are only effective at unusually high therapeutic dosages or cause serious side effects or immune suppression [248]. A few studies have investigated the potential of rCoVs with a mutated E or lacking E, specifically focussing on SARS- and MERS-CoV, as live attenuated vaccine candidates with some promising results [34, 36, 165, 249, 250]. Vaccinated animal models developed robust immune responses, both cellular and humoral, and were protected against infective challenges. This shows that CoV vaccines with mutated or deficient in E can potentially be used for prophylactic treatment, but the duration of immunity does not seem to have been established yet.

**Fig. 6:**
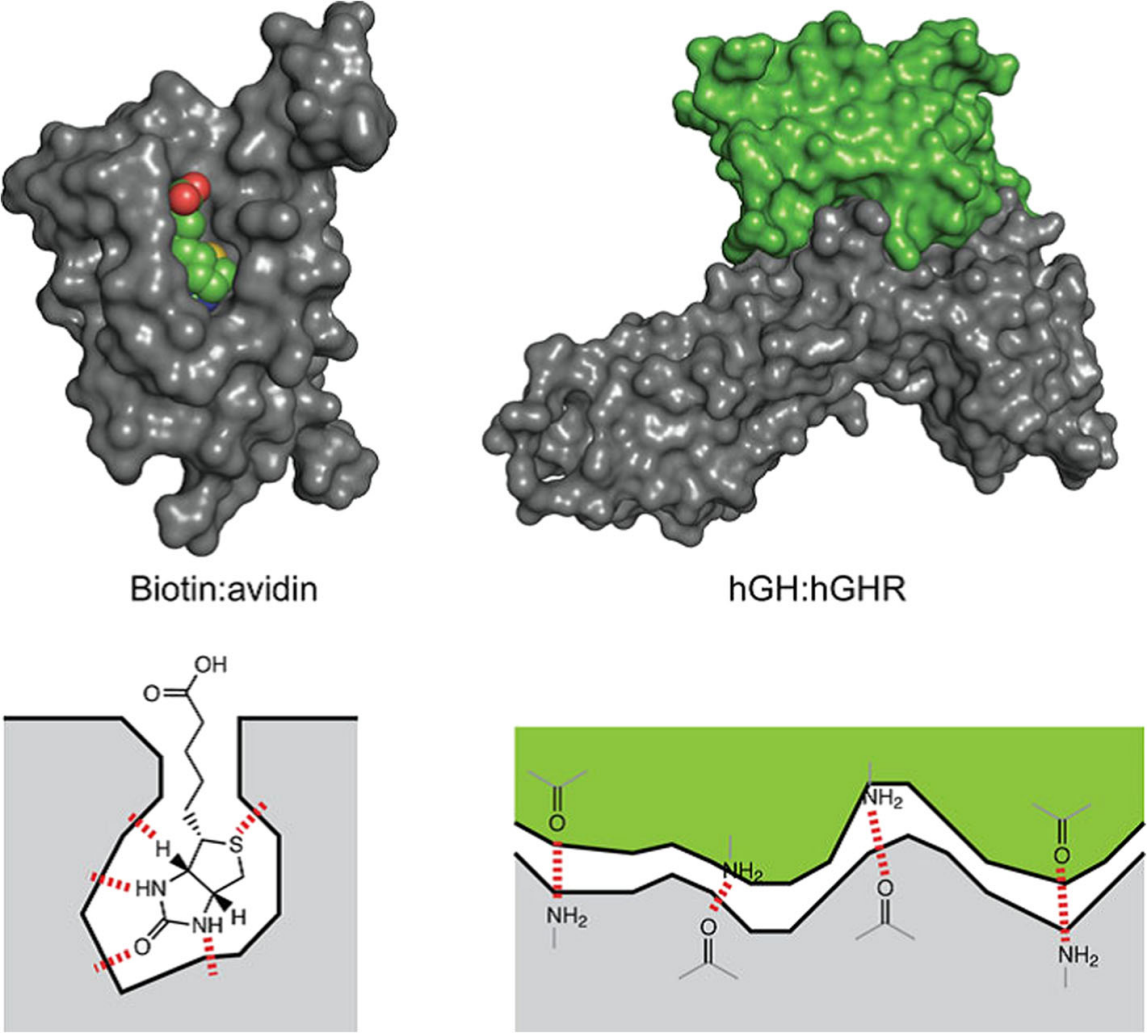
Mechanisms of interaction between small molecules and proteins, and protein-protein interactions. Left: The binding of biotin to avidin occurs in a deep groove, while the interaction between the human growth hormone (hGH) and the hGH receptor (hGHR) occurs over a larger, flatter area [254]

Viruses exploit the extensive network of their host cell’s signalling pathways to promote viral replication and propagation [251, 252]. This dependence on PPIs offers the unique opportunity to target both viral-host and intraviral PPIs and, thereby, stop viral replication and propagation. Therapies that use small-molecule drugs have the advantage of small size, which allows the drugs to cross cell membranes efficiently, but it also severely limits the selectivity and targeting capabilities of the drug, which often leads to undesired side-effects [253]. Interactions between proteins take place over large, flat surface areas that feature shallow interaction sites. Small-molecule drugs, however, tend to bind to deep grooves or hydrophobic pockets not always found on the surface of target proteins, making it difficult for such drugs to disrupt PPIs (Fig. 6) [253–255]. Larger, protein-based therapies, on the other hand, make use of insulin, growth factors, and engineered antibodies, that form many more, and much stronger, interactions, making these therapies more potent and selective for their targets. Such properties result in fewer side-effects but the size of these agents also restricts their ability to cross the membranes of target cells [253]. This calls for therapeutic agents that can bridge the gap between molecules that are large enough to be specific and potent for their targets but still small enough to be able to cross target cell membranes efficiently and can also be manufactured easily.

Stapled peptides fulfil these criteria to a large extent and have been applied to various human diseases and fields such as cancer, infections, metabolism, neurology, and endocrinology [256–260]. In fact, Aileron Therapeutics have already developed two stapled peptides, ALRN-5281 and ATSP-7041. The company has already completed the first-in-human trail with ALRN-5281 for the treatment of rare endocrine diseases, such as adult growth hormone deficiency. Moreover, ATSP-7041 was designed to target intracellular PPIs, specifically murine double minute 2 (MDM2) and murine double minute X (MDMX) [261]. To the best of the author’s knowledge, only a few studies so far have investigated the potential of stapled peptides as antiviral agents, with promising results for both intracellular and extracellular targets. The focus so far has only been on HIV-1, RSV, and HCV [260, 262–265].

Granted, the therapeutic application of stapled peptides, particularly regarding viral infections, is still relatively new, but their numerous advantages give them tremendous potential as antiviral agents. Stapled peptides (1) can inhibit PPIs; (2) are more specific for their targets than small-molecule drugs, which also decreases the risk of unwanted side-effects; (3) can target diseases that are otherwise difficult to treat, referred to as “undruggable”; (4) can be modified easily to enhance membrane permeability, potency, and half-life; (5) have a short market time [253, 266, 267]. As more viral PPIs for CoV E are identified, the repertoire of stapled peptide targets also expands making it easier to limit viral replication, propagation, and even pathogenesis. Stapled peptides have the potential to be used as antiviral agents that can work effectively at multiple levels.

Autophagy is a cellular process that recycles excess or damaged cellular material to maintain the energy levels of the cell and ensure its survival. The material is removed from the cytoplasm by forming enclosed DMVs known as autophagosomes and then fused with lysosomes to be degraded [268, 269]. Recent studies have increasingly pointed to the involvement of autophagy components in viral infections [270]. Some suggest that it might have an antiviral function by inhibiting viral replication [271–273]. Others reported inhibition or subversion of autophagy as a defence mechanism to promote viral propagation [274–276]. Others still, notably RNA viruses, appear to exploit autophagy for the purpose of viral propagation [277, 278]. Regarding CoVs, replication of TGEV is negatively regulated by autophagy [279]. Interestingly, PRRSV activates autophagy machinery, possibly to enhance viral replication as certain components of autophagy are required for MHV replication [280, 281]. These studies suggest the possibility of CoVs exploiting autophagy for replicative purposes. It has even been proposed that the DMVs formed in CoV-infected cells might be the result of autophagy and derived from the rough ER [281]. Recently, an increase in cytosolic Ca^2+^, presumably from the ER lumen, has been implicated in autophagy induction by protein 2B (P2B) of the foot and mouth disease virus (FMDV) [282]. The rotavirus non-structural protein 4 (NSP4) reportedly induces autophagy by a similar mechanism [283]. Considering these studies, along with the ability of SARS-CoV to channel Ca^2+^, it is not inconceivable that CoV E viroporin could induce autophagy in CoV-infected cells by increasing cytosolic Ca^2+^. However, experimental evidence would be required to support the possibility of such a mechanism in CoVs.

#### The multifunctional role CoV E protein: A central role in assembly, release, and pathogenesis?

From studies, it appears that some viral proteins do not have unique, definitive functions. Despite the deletion of some viral genes, the viral life cycle continues, suggesting that other viral genes can compensate for this loss. It was recently shown to be the case for the vaccinia virus [284]. This is also evident in the varied requirements of the E protein for different CoVs and the reason(s) for this is not understood. Trafficking and maturation of TGEV virions is arrested without E [40]. Virions of MHV ΔE are capable of producing viable, replicating progeny [39]. Deletion of E from SARS-CoV attenuates the virus whereas, in the case of MERS-CoV, virions are propagation deficient [35, 165]. Certain CoV accessory proteins appear to be able to complement, or sometimes even compensate for, the absence of E in processes such as assembly, release, and the pathogenesis of some CoVs [30]*.* It is particularly noteworthy that SARS-CoV encodes two accessory proteins, 3a and 8a, that might exhibit relative compensatory functions in the absence of E [285, 286]. In terms of viral replication in vivo and in vitro, 3a could partially compensate for the loss of E. Moreover, 3a also contains a PBM and might be able to compensate for the loss of E to an extent but utilises different signalling pathways [285]. Although the study demonstrated that even the accessory proteins demonstrate some measure of dispensability, the virus still encodes these additional proteins with overlapping functions. The dynamics between these proteins, however, are not quite clear yet and warrants further investigation. What is clear, though, is that viroporin proteins, case in point IAV M2, can exhibit a multitude of different functions independent of their ion-channel properties [153, 184]. The studies in this review have shown that CoV E could be involved in multiple aspects of the viral replication cycle: from assembly and induction of membrane curvature to scission or budding and release to apoptosis, inflammation and even autophagy. Although a lot of progress has been made on CoV E, there is still much to be discovered about this small, enigmatic protein.

## Abbreviations

A15D: Alanine residue 15 mutated to aspartic acid
A26F: Alanine residue 26 mutated to phenylalanine
altPBM: alanine mutated PBM
ARDS: Acute respiratory distress syndrome
Bcl-xL: B-cell lymphoma-extra-large
BCoV: Bat coronavirus
Ca^2+^: Calcium ion
CCoV: Canine coronavirus
Cl^−^: Chloride ion
CMs: Convoluted membranes
CoV(s): Coronavirus (es)
C-terminus: Carboxy terminus
Dlg1: *Drosophila* disc large tumour \ressor
DMVs: Double-membrane vesicles
E: Envelope protein
EM: Electron microscopy
Env: Envelope glycoprotein gp160
ER: Endoplasmic reticulum
ERAD: ER-assisted degradation
ERGIC: Endoplasmic reticulum Golgi intermediate compartment
ESCRT: Endosomal sorting complex required for transport
F13 L: vaccinia virus envelope phospholipase F13 protein
F20 L: phenylalanine residue 20 mutated to leucine
F25D: phenylalanine residue 20 mutated to aspartic acid
F26 L: phenylalanine residue 26 mutated to leucine
FeCoV: feline coronavirus
FMDV: foot and mouth disease virus
GFP: Green fluorescent protein
GST: Glutathione-S-transferase
H^+^: Hydrogen ion
HA: Haemagglutinin
HBV: Hepatitis B virus
HCoV(s): Human coronavirus (es)
HCoV-229E: Human coronavirus 229E
HCoV-4408: Human coronavirus 4408
HCoV-HKU1: Human coronavirus HKU1
HCoV-NL63: Human coronavirus NL63
HCoV-OC43: Human coronavirus OC43
HCV: Hepatitis C virus
HD: Hydrophobic domain
HEV: porcine hemagglutinating encephalomyelitis virus
hGH: human growth hormone
hGHR: human growth hormone receptor
HIV: human immunodeficiency virus
IAV: Influenza A virus
IBV: avian infectious bronchitis virus
K^+^: potassium ion
kb: kilobases
kDa: kilodalton
L19A: Leucine residue 19 mutated to alanine
L27S: Leucine residue 27 mutated to serine
L37R: Leucine residue 37 mutated to arginine
LRTIs: Lower respiratory tract infections
M: Membrane protein
M2: Matrix-2 protein
MAPK: Mitogen-activated protein kinase
MDM2: Murine double minute 2
MDMX: Murine double minute X
MERS: Middle-East respiratory syndrome
MERS-CoV: Middle-East respiratory syndrome coronavirus
MHC-I: major histocompatibility complex I
MHV: Murine hepatitis virus
MS: Mass spectrometry
mutPBM: glycine mutated PBM
N: Nucleocapsid protein
N15A: asparagine residue 15 mutated to alanine
N5, 15, 48, 66: asparagine residues 5, 15, 48, 66
Na^+^: sodium ion
Nef: negative regulatory factor
NLRP3: NOD-like receptor family, pyrin domain containing 3
Nsp(s) 3, 4, 6: non-structural protein(s) 3, 4, 6
NSP4: Non-structural protein 4
N-terminus: amino terminus
P2B: protein 2B
PALS1: Protein associated with *Caenorhabditis elegans* lin-7 protein 1
PBM: PDZ-binding motif
PDZ: Postsynaptic density protein 95 (PSD95)/*Drosophila* disc large tumour suppressor (Dlg1)/zonula occludens-1 protein (zo-1)
PEDV: Porcine epidemic diarrhoea coronavirus
PPI(s): Protein-protein interaction(s)
PRCoV: Porcine respiratory coronavirus
PRRSV: Porcine reproductive and respiratory syndrome virus
PSD95: Postsynaptic density protein 95
rCoVs: recombinant coronaviruses
RNA: Ribonucleic acid
RSV: Respiratory syncytial virus
S: Spike protein
SARS: Severe acute respiratory syndrome
SARS-CoV: severe acute respiratory syndrome coronavirus
Sf9: *Spodoptera frugiperda* cell line
SH: Small hydrophobic
SIV: Simian immunodeficiency virus
T16A: Threonine residue 16 mutated to alanine
T30I: Threonine residue 30 mutated to isoleucine
TAP: Tandem affinity purification
TAP-MS: Tandem affinity purification coupled with mass spectrometry
TCoV: Turkey coronavirus
TGEV: Transmissible gastroenteritis coronavirus
TMD: Transmembrane domain
UPR: Unfolded-protein response
URTIs: Upper respiratory tract infections
V25: Valine residue 25
V25F: Valine residue 25 mutated to phenylalanine
VLP(s): Virus-like particle(s)
zo-1: zonula occludens-1 protein
α: alpha
β: beta
γ: gamma
Δ6: recombinant SARS-CoV deletion mutant number 6
ΔE: deleted E gene
ΔPBM: recombinant SARS-CoV mutant with deleted PBM

## References

[1] van Regenmortel MHV, Fauquet CM, Bishop DHL, Carstens EB, Estes MK, Lemon SM, et al. Coronaviridae. In: MHV v R, Fauquet CM, DHL B, Carstens EB, Estes MK, Lemon SM, et al., editors. Virus taxonomy: Classification and nomenclature of viruses Seventh report of the International Committee on Taxonomy of Viruses. San Diego: Academic Press; 2000. p. 835–49. ISBN 0123702003.

[2] Pradesh U, Upadhayay PDD, Vigyan PC (2014). Coronavirus infection in equines: A review. Asian J Anim Vet Adv.

[3] Lee C (2015). Porcine epidemic diarrhea virus: An emerging and re-emerging epizootic swine virus. Virol J.

[4] Bande Faruku, Arshad Siti Suri, Hair Bejo Mohd, Moeini Hassan, Omar Abdul Rahman (2015). Progress and Challenges toward the Development of Vaccines against Avian Infectious Bronchitis. Journal of Immunology Research.

[5] Owusu M, Annan A, Corman VM, Larbi R, Anti P, Drexler JF (2014). Human coronaviruses associated with upper respiratory tract infections in three rural areas of Ghana. PLoS One.

[6] van der Hoek L (2007). Human coronaviruses: What do they cause? Antiviral Therapy.

[7] Vabret A, Mourez T, Gouarin S, Petitjean J, Freymuth F (2003). An outbreak of coronavirus OC43 respiratory infection in Normandy, France. Clin Infect Dis.

[8] Gerna G, Campanini G, Rovida F, Percivalle E, Sarasini A, Marchi A (2006). Genetic variability of human coronavirus OC43-, 229E-, and NL63-like strains and their association with lower respiratory tract infections of hospitalized infants and immunocompromised patients. J Med Virol.

[9] Vabret A, Dina J, Gouarin S, Petitjean J, Tripey V, Brouard J (2008). Human (non-severe acute respiratory syndrome) coronavirus infections in hospitalised children in France. J Paediatr Child Health.

[10] Gerna G, Percivalle E, Sarasini A, Campanini G, Piralla A, Rovida F (2007). Human respiratory coronavirus HKU1 versus other coronavirus infections in Italian hospitalised patients. J Clin Virol.

[11] Fouchier RA, Kuiken T, Schutten M, Van Amerongen G, van Doornum GJ, van den Hoogen BG (2003). Aetiology: Koch's postulates fulfilled for SARS virus. Nature..

[12] Mäkelä MJ, Puhakka T, Ruuskanen O, Leinonen M, Saikku P, Kimpimäki M (1998). Viruses and bacteria in the etiology of the common cold. J Clin Microbiol.

[13] Zhong N, Zheng B, Li Y, Poon L, Xie Z, Chan K (2003). Epidemiology and cause of severe acute respiratory syndrome (SARS) in Guangdong, People's Republic of China, in February, 2003. Lancet.

[14] Woo PC, Lau SK, Huang Y, Yuen K-Y (2009). Coronavirus diversity, phylogeny and interspecies jumping. Exp Biol Med.

[15] van Elden LJ, Anton MAM, van Alphen F, Hendriksen KA, Hoepelman AI, van Kraaij MG (2004). Frequent detection of human coronaviruses in clinical specimens from patients with respiratory tract infection by use of a novel real-time reverse-transcriptase polymerase chain reaction. J Infect Dis.

[16] Kim KY, Han SY, Kim H-S, Cheong H-M, Kim SS, Kim DS (2017). Human coronavirus in the 2014 winter season as a cause of lower respiratory tract infection. Yonsei Med J.

[17] Dominguez SR, Robinson CC, Holmes KV (2009). Detection of four human coronaviruses in respiratory infections in children: A one-year study in Colorado. J Med Virol.

[18] Jimenez-Guardeño JM, Nieto-Torres JL, DeDiego ML, Regla-Nava JA, Fernandez-Delgado R, Castaño-Rodriguez C (2014). The PDZ-binding motif of severe acute respiratory syndrome coronavirus envelope protein is a determinant of viral pathogenesis. PLoS Pathog.

[19] Lau SK, Woo PC, Li KS, Huang Y, Tsoi H-W, Wong BH (2005). Severe acute respiratory syndrome coronavirus-like virus in Chinese horseshoe bats. Proc Natl Acad Sci.

[20] Rest JS, Mindell DP (2003). SARS associated coronavirus has a recombinant polymerase and coronaviruses have a history of host-shifting. Infect Genet Evol.

[21] Lu G, Wang Q, Gao GF (2015). Bat-to-human: Spike features determining ‘host jump’of coronaviruses SARS-CoV, MERS-CoV, and beyond. Trends Microbiol.

[22] Chan JF-W, Tse H, Jin D-Y, Yuen K-Y, To KK-W (2013). Interspecies transmission and emergence of novel viruses: Lessons from bats and birds. Trends Microbiol.

[23] Hon C-C, Lam T-Y, Shi Z-L, Drummond AJ, Yip C-W, Zeng F (2008). Evidence of the recombinant origin of a bat severe acute respiratory syndrome (SARS)-like coronavirus and its implications on the direct ancestor of SARS coronavirus. J Virol.

[24] World Health Organization WHO. Summary of probable SARS cases with onset of illness from 1 November 2002 to 31 July 2003 2003. Available from: http://www.who.int/csr/sars/country/table2004_04_21/en/index.html.

[25] World Health Organization WHO. WHO MERS-CoV Global Summary and Assessment of Risk, August 2018 (WHO/MERS/RA/August18) 2018. Available from: http://www.who.int/csr/disease/coronavirus_infections/risk-assessment-august-2018.pdf?ua=1.

[26] Lou Z, Sun Y, Rao Z (2014). Current progress in antiviral strategies. Trends Pharmacol Sci.

[27] Kilianski A, Baker SC (2014). Cell-based antiviral screening against coronaviruses: Developing virus-specific and broad-spectrum inhibitors. Antivir Res.

[28] Kilianski A, Mielech A, Deng X, Baker SC. Assessing activity and inhibition of MERS-CoV papain-like and 3C-like proteases using luciferase-based biosensors. J Virol. 2013;66:JVI. 02105–02113. 10.1128/JVI.02105-13 PMC3807373 23986593

[29] Masters PS (2006). The molecular biology of coronaviruses. Adv Virus Res.

[30] Liu DX, Fung TS, Chong KK-L, Shukla A, Hilgenfeld R (2014). Accessory proteins of SARS-CoV and other coronaviruses. Antivir Res.

[31] Heald-Sargent T, Gallagher T (2012). Ready, set, fuse! The coronavirus spike protein and acquisition of fusion competence. Viruses..

[32] Graham RL, Becker MM, Eckerle LD, Bolles M, Denison MR, Baric RS (2012). A live, impaired-fidelity coronavirus vaccine protects in an aged, immunocompromised mouse model of lethal disease. Nat Med.

[33] Enjuanes L, Nieto-Torres JL, Jimenez-Guardeño JM, DeDiego ML, Dormitzer P, Mandl CW, Rappuoli R (2011). Recombinant live vaccines to protect against the severe acute respiratory syndrome coronavirus. Replicating vaccines, Birkhauser advances in infectious diseases book series (BAID).

[34] Regla-Nava JA, Nieto-Torres JL, Jimenez-Guardeño JM, Fernandez-Delgado R, Fett C, Castaño-Rodríguez C (2015). SARS coronaviruses with mutations in E protein are attenuated and promising vaccine candidates. J Virol.

[35] DeDiego ML, Álvarez E, Almazán F, Rejas MT, Lamirande E, Roberts A (2007). A severe acute respiratory syndrome coronavirus that lacks the E gene is attenuated *in vitro* and *in vivo*. J Virol.

[36] Netland J, DeDiego ML, Zhao J, Fett C, Álvarez E, Nieto-Torres JL (2010). Immunization with an attenuated severe acute respiratory syndrome coronavirus deleted in E protein protects against lethal respiratory disease. Virology..

[37] Mortola E, Roy P (2004). Efficient assembly and release of SARS coronavirus-like particles by a heterologous expression system. FEBS Lett.

[38] Wang C, Zheng X, Gai W, Zhao Y, Wang H, Wang H (2017). MERS-CoV virus-like particles produced in insect cells induce specific humoural and cellular immunity in rhesus macaques. Oncotarget..

[39] Kuo L, Masters PS (2003). The small envelope protein E is not essential for murine coronavirus replication. J Virol.

[40] Ortego J, Ceriani JE, Patiño C, Plana J, Enjuanes L (2007). Absence of E protein arrests transmissible gastroenteritis coronavirus maturation in the secretory pathway. Virology..

[41] Ruch TR, Machamer CE (2012). The coronavirus E protein: Assembly and beyond. Viruses..

[42] Siu Y, Teoh K, Lo J, Chan C, Kien F, Escriou N (2008). The M, E, and N structural proteins of the severe acute respiratory syndrome coronavirus are required for efficient assembly, trafficking, and release of virus-like particles. J Virol.

[43] Kirchdoerfer RN, Cottrell CA, Wang N, Pallesen J, Yassine HM, Turner HL (2016). Pre-fusion structure of a human coronavirus spike protein. Nature..

[44] Song HC, Seo M-Y, Stadler K, Yoo BJ, Choo Q-L, Coates SR (2004). Synthesis and characterization of a native, oligomeric form of recombinant severe acute respiratory syndrome coronavirus spike glycoprotein. J Virol.

[45] Fehr AR, Perlman S (2015). Coronaviruses: An overview of their replication and pathogenesis.

[46] Glowacka I, Bertram S, Müller MA, Allen P, Soilleux E, Pfefferle S (2011). Evidence that TMPRSS2 activates the severe acute respiratory syndrome coronavirus spike protein for membrane fusion and reduces viral control by the humoral immune response. J Virol.

[47] Qian Z, Dominguez SR, Holmes KV (2013). Role of the spike glycoprotein of human Middle East respiratory syndrome coronavirus (MERS-CoV) in virus entry and syncytia formation. PLoS One.

[48] de Haan CA, Rottier PJ (2005). Molecular interactions in the assembly of coronaviruses. Adv Virus Res.

[49] McBride R, van Zyl M, Fielding BC (2014). The coronavirus nucleocapsid is a multifunctional protein. Viruses..

[50] Tooze J, Tooze S, Warren G (1984). Replication of coronavirus MHV-A59 in sac-cells: Determination of the first site of budding of progeny virions. Eur J Cell Biol.

[51] Klumperman J, Locker JK, Meijer A, Horzinek MC, Geuze HJ, Rottier P (1994). Coronavirus M proteins accumulate in the Golgi complex beyond the site of virion budding. J Virol.

[52] Boscarino JA, Logan HL, Lacny JJ, Gallagher TM (2008). Envelope protein palmitoylations are crucial for murine coronavirus assembly. J Virol.

[53] Ruch TR, Machamer CE (2011). The hydrophobic domain of infectious bronchitis virus E protein alters the host secretory pathway and is important for release of infectious virus. J Virol.

[54] Neuman BW, Kiss G, Kunding AH, Bhella D, Baksh MF, Connelly S (2011). A structural analysis of M protein in coronavirus assembly and morphology. J Struct Biol.

[55] de Haan CA, Vennema H, Rottier PJ (2000). Assembly of the coronavirus envelope: homotypic interactions between the M proteins. J Virol.

[56] Lim K, Liu D (2001). The missing link in coronavirus assembly: retention of the avian coronavirus infectious bronchitis virus envelope protein in the pre-Golgi compartments and physical interaction between the envelope and membrane proteins. J Biol Chem.

[57] Opstelten DJ, Raamsman M, Wolfs K, Horzinek MC, Rottier P (1995). Envelope glycoprotein interactions in coronavirus assembly. J Cell Biol.

[58] Escors D, Ortego J, Laude H, Enjuanes L (2001). The membrane M protein carboxy terminus binds to transmissible gastroenteritis coronavirus core and contributes to core stability. J Virol.

[59] Narayanan K, Maeda A, Maeda J, Makino S (2000). Characterization of the coronavirus M protein and nucleocapsid interaction in infected cells. J Virol.

[60] Corse E, Machamer CE (2000). Infectious bronchitis virus E protein is targeted to the Golgi complex and directs release of virus-like particles. J Virol.

[61] Corse E, Machamer CE (2003). The cytoplasmic tails of infectious bronchitis virus E and M proteins mediate their interaction. Virology..

[62] Bos EC, Luytjes W, van der Meulen H, Koerten HK, Spaan WJ (1996). The production of recombinant infectious DI-particles of a murine coronavirus in the absence of helper virus. Virology..

[63] Vennema H, Godeke G-J, Rossen J, Voorhout W, Horzinek M, Opstelten D (1996). Nucleocapsid-independent assembly of coronavirus-like particles by co-expression of viral envelope protein genes. EMBO J.

[64] Baudoux P, Carrat C, Besnardeau L, Charley B, Laude H (1998). Coronavirus pseudoparticles formed with recombinant M and E proteins induce alpha interferon synthesis by leukocytes. J Virol.

[65] Venkatagopalan P, Daskalova SM, Lopez LA, Dolezal KA, Hogue BG (2015). Coronavirus envelope (E) protein remains at the site of assembly. Virology..

[66] Nieto-Torres JL, DeDiego ML, Álvarez E, Jiménez-Guardeño JM, Regla-Nava JA, Llorente M (2011). Subcellular location and topology of severe acute respiratory syndrome coronavirus envelope protein. Virology..

[67] Curtis KM, Yount B, Baric RS (2002). Heterologous gene expression from transmissible gastroenteritis virus replicon particles. J Virol.

[68] Ortego J, Escors D, Laude H, Enjuanes L (2002). Generation of a replication-competent, propagation-deficient virus vector based on the transmissible gastroenteritis coronavirus genome. J Virol.

[69] Kuo L, Hurst KR, Masters PS (2007). Exceptional flexibility in the sequence requirements for coronavirus small envelope protein function. J Virol.

[70] Arbely E, Khattari Z, Brotons G, Akkawi M, Salditt T, Arkin IT (2004). A highly unusual palindromic transmembrane helical hairpin formed by SARS coronavirus E protein. J Mol Biol.

[71] Raamsman MJ, Locker JK, de Hooge A, de Vries AA, Griffiths G, Vennema H (2000). Characterization of the coronavirus mouse hepatitis virus strain A59 small membrane protein E. J Virol.

[72] Li Y, Surya W, Claudine S, Torres J (2014). Structure of a conserved Golgi complex-targeting signal in coronavirus envelope proteins. J Biol Chem.

[73] Liao Y, Yuan Q, Torres J, Tam J, Liu D (2006). Biochemical and functional characterization of the membrane association and membrane permeabilizing activity of the severe acute respiratory syndrome coronavirus envelope protein. Virology..

[74] Surya Wahyu, Samso Montserrat, Torres Jaume (2013). Structural and Functional Aspects of Viroporins in Human Respiratory Viruses: Respiratory Syncytial Virus and Coronaviruses. Respiratory Disease and Infection - A New Insight.

[75] Torres J, Maheswari U, Parthasarathy K, Ng L, Liu DX, Gong X (2007). Conductance and amantadine binding of a pore formed by a lysine-flanked transmembrane domain of SARS coronavirus envelope protein. Protein Sci.

[76] Verdiá-Báguena C, Nieto-Torres JL, Alcaraz A, DeDiego ML, Torres J, Aguilella VM (2012). Coronavirus E protein forms ion channels with functionally and structurally-involved membrane lipids. Virology..

[77] Nieto-Torres JL, DeDiego ML, Verdiá-Báguena C, Jimenez-Guardeño JM, Regla-Nava JA, Fernandez-Delgado R (2014). Severe acute respiratory syndrome coronavirus envelope protein ion channel activity promotes virus fitness and pathogenesis. PLoS Pathog.

[78] Verdiá-Báguena C, Nieto-Torres JL, Alcaraz A, DeDiego ML, Enjuanes L, Aguilella VM (2013). Analysis of SARS-CoV E protein ion channel activity by tuning the protein and lipid charge. Biochim Biophys Acta.

[79] Wu Q, Zhang Y, Lü H, Wang J, He X, Liu Y (2003). The E protein is a multifunctional membrane protein of SARS-CoV. Genomics, Proteomics & Bioinformatics.

[80] Du Y, Zuckermann FA, Yoo D (2010). Myristoylation of the small envelope protein of porcine reproductive and respiratory syndrome virus is non-essential for virus infectivity but promotes its growth. Virus Res.

[81] Cohen JR, Lin LD, Machamer CE (2011). Identification of a Golgi targeting signal in the cytoplasmic tail of the severe acute respiratory syndrome coronavirus envelope protein. J Virol.

[82] Teoh K-T, Siu Y-L, Chan W-L, Schlüter MA, Liu C-J, Peiris JM (2010). The SARS coronavirus E protein interacts with PALS1 and alters tight junction formation and epithelial morphogenesis. Mol Biol Cell.

[83] Javier RT, Rice AP (2011). Emerging theme: cellular PDZ proteins as common targets of pathogenic viruses. J Virol.

[84] Hung AY, Sheng M (2002). PDZ domains: structural modules for protein complex assembly. J Biol Chem.

[85] Münz M, Hein J, Biggin PC (2012). The role of flexibility and conformational selection in the binding promiscuity of PDZ domains. PLoS Comput Biol.

[86] Gerek ZN, Keskin O, Ozkan SB (2009). Identification of specificity and promiscuity of PDZ domain interactions through their dynamic behavior. Proteins Struct Funct Bioinf.

[87] Yang Y, Xiong Z, Zhang S, Yan Y, Nguyen J, Ng B (2005). Bcl-xL inhibits T-cell apoptosis induced by expression of SARS coronavirus E protein in the absence of growth factors. Biochem J.

[88] Jimenez-Guardeño JM, Regla-Nava JA, Nieto-Torres JL, DeDiego ML, Castaño-Rodriguez C, Fernandez-Delgado R (2015). Identification of the mechanisms causing reversion to virulence in an attenuated SARS-CoV for the design of a genetically stable vaccine. PLoS Pathog.

[89] Hogue BG, Machamer CE (2008). Coronavirus structural proteins and virus assembly.

[90] Westerbeck Jason W., Machamer Carolyn E. (2015). A Coronavirus E Protein Is Present in Two Distinct Pools with Different Effects on Assembly and the Secretory Pathway. Journal of Virology.

[91] Yuan Q, Liao Y, Torres J, Tam J, Liu D (2006). Biochemical evidence for the presence of mixed membrane topologies of the severe acute respiratory syndrome coronavirus envelope protein expressed in mammalian cells. FEBS Lett.

[92] Nal B, Chan C, Kien F, Siu L, Tse J, Chu K (2005). Differential maturation and subcellular localization of severe acute respiratory syndrome coronavirus surface proteins S, M and E. J Gen Virol.

[93] Corse E, Machamer CE (2002). The cytoplasmic tail of infectious bronchitis virus E protein directs Golgi targeting. J Virol.

[94] Maeda J, Repass JF, Maeda A, Makino S (2001). Membrane topology of coronavirus E protein. Virology..

[95] Godet M, L'Haridon R, Vautherot J-F, Laude H (1992). TGEV coronavirus ORF4 encodes a membrane protein that is incorporated into virions. Virology..

[96] Hofmann K (1993). TMbase-A database of membrane spanning proteins segments. Biol Chem Hoppe Seyler.

[97] Tusnady GE, Simon I (1998). Principles governing amino acid composition of integral membrane proteins: application to topology prediction1. J Mol Biol.

[98] Krogh A, Larsson B, Von Heijne G, Sonnhammer EL (2001). Predicting transmembrane protein topology with a hidden Markov model: application to complete genomes. J Mol Biol.

[99] Jones DT (2007). Improving the accuracy of transmembrane protein topology prediction using evolutionary information. Bioinformatics..

[100] Nugent T, Jones DT (2009). Transmembrane protein topology prediction using support vector machines. BMC Bioinformatics.

[101] Elofsson A (2007). Heijne Gv. Membrane protein structure: prediction versus reality. Annu Rev Biochem.

[102] Birzele F, Kramer S (2006). A new representation for protein secondary structure prediction based on frequent patterns. Bioinformatics..

[103] Chen K, Kurgan L, Ruan J, editors. Optimization of the sliding window size for protein structure prediction. In: 2006 IEEE Symposium on Computational Intelligence and Bioinformatics and Computational Biology: IEEE; 2006. 10.1109/CIBCB.2006.330959.

[104] Zviling M, Leonov H, Arkin IT (2005). Genetic algorithm-based optimization of hydrophobicity tables. Bioinformatics..

[105] Schlessinger A, Rost B (2005). Protein flexibility and rigidity predicted from sequence. Proteins Struct Funct Bioinf.

[106] Jones DT (1999). Protein secondary structure prediction based on position-specific scoring matrices. J Mol Biol.

[107] Bodén M, Yuan Z, Bailey TL (2006). Prediction of protein continuum secondary structure with probabilistic models based on NMR solved structures. BMC Bioinformatics.

[108] Sander O, Sommer I, Lengauer T (2006). Local protein structure prediction using discriminative models. BMC Bioinformatics.

[109] Ruch TR, Machamer CE (2012). A single polar residue and distinct membrane topologies impact the function of the infectious bronchitis coronavirus E protein. PLoS Pathog.

[110] Rocks O, Peyker A, Kahms M, Verveer PJ, Koerner C, Lumbierres M (2005). An acylation cycle regulates localization and activity of palmitoylated Ras isoforms. Science..

[111] Basu J (2004). Protein palmitoylation and dynamic modulation of protein function. Curr Sci.

[112] Salaun C, Greaves J, Chamberlain LH (2010). The intracellular dynamic of protein palmitoylation. J Cell Biol.

[113] Fujiwara Y, Kondo HX, Shirota M, Kobayashi M, Takeshita K, Nakagawa A (2016). Structural basis for the membrane association of ankyrinG via palmitoylation. Sci Rep.

[114] Sobocińska J, Roszczenko-Jasińska P, Ciesielska A, Kwiatkowska K (2018). Protein Palmitoylation and its Role in Bacterial and viral infections. Front Immunol.

[115] Grosenbach DW, Ulaeto DO, Hruby DE (1997). Palmitylation of the vaccinia virus 37-kDa major envelope antigen identification of a conserved acceptor motif and biological relevance. J Biol Chem.

[116] Majeau Nathalie, Fromentin Rémi, Savard Christian, Duval Marie, Tremblay Michel J., Leclerc Denis (2009). Palmitoylation of Hepatitis C Virus Core Protein Is Important for Virion Production. Journal of Biological Chemistry.

[117] Lopez LA, Riffle AJ, Pike SL, Gardner D, Hogue BG (2008). Importance of conserved cysteine residues in the coronavirus envelope protein. J Virol.

[118] Resh MD (1999). Fatty acylation of proteins: new insights into membrane targeting of myristoylated and palmitoylated proteins. Biochim Biophys Acta.

[119] He M, Jenkins P, Bennett V (2012). Cysteine 70 of ankyrin-G is S-palmitoylated and is required for function of ankyrin-G in membrane domain assembly. J Biol Chem.

[120] Wilcox C, Hu J-S, Olson EN (1987). Acylation of proteins with myristic acid occurs cotranslationally. Science..

[121] James G, Olson EN (1990). Fatty acylated proteins as components of intracellular signaling pathways. Biochemistry..

[122] Boutin JA (1997). Myristoylation. Cell Signal.

[123] Nimchuk Z, Marois E, Kjemtrup S, Leister RT, Katagiri F, Dangl JL (2000). Eukaryotic fatty acylation drives plasma membrane targeting and enhances function of several type III effector proteins from Pseudomonas syringae. Cell..

[124] Chow M, Newman J, Filman D, Hogle J, Rowlands D, Brown F (1987). Myristylation of picornavirus capsid protein VP4 and its structural significance. Nature..

[125] Henderson L, Benveniste R, Sowder R, Copeland T, Schultz A, Oroszlan S (1988). Molecular characterization of gag proteins from simian immunodeficiency virus (SIVMne). J Virol.

[126] Harris M, Hislop S, Patsilinacos P, Neil JC (1992). *In vivo* derived HIV-1 nef gene products are heterogeneous and lack detectable nucleotide binding activity. AIDS Res Hum Retrovir.

[127] Persing DH, Varmus H, Ganem D (1987). The preS1 protein of hepatitis B virus is acylated at its amino terminus with myristic acid. J Virol.

[128] Álvarez E, DeDiego ML, Nieto-Torres JL, Jiménez-Guardeño JM, Marcos-Villar L, Enjuanes L (2010). The envelope protein of severe acute respiratory syndrome coronavirus interacts with the non-structural protein 3 and is ubiquitinated. Virology..

[129] Isaacson MK, Ploegh HL (2009). Ubiquitination, ubiquitin-like modifiers, and deubiquitination in viral infection. Cell Host Microbe.

[130] Keng C-T, Åkerström S, Leung CS-W, Poon LL, Peiris JM, Mirazimi A (2011). SARS coronavirus 8b reduces viral replication by down-regulating E via an ubiquitin-independent proteasome pathway. Microbes Infect.

[131] Vigerust DJ, Shepherd VL (2007). Virus glycosylation: role in virulence and immune interactions. Trends Microbiol.

[132] Fung TS, Liu DX (2018). Post-translational modifications of coronavirus proteins: roles and function. Futur Virol.

[133] Nilsson I, Von Heijne G (1993). Determination of the distance between the oligosaccharyltransferase active site and the endoplasmic reticulum membrane. J Biol Chem.

[134] Wang Bin, Wang Yujie, Frabutt Dylan A., Zhang Xihe, Yao Xiaoyu, Hu Dan, Zhang Zhuo, Liu Chaonan, Zheng Shimin, Xiang Shi-Hua, Zheng Yong-Hui (2017). Mechanistic understanding ofN-glycosylation in Ebola virus glycoprotein maturation and function. Journal of Biological Chemistry.

[135] Parthasarathy K, Ng L, Lin X, Liu DX, Pervushin K, Gong X (2008). Structural flexibility of the pentameric SARS coronavirus envelope protein ion channel. Biophys J.

[136] Pervushin K, Tan E, Parthasarathy K, Lin X, Jiang FL, Yu D (2009). Structure and inhibition of the SARS coronavirus envelope protein ion channel. PLoS Pathog.

[137] Torres J, Wang J, Parthasarathy K, Liu DX (2005). The transmembrane oligomers of coronavirus protein E. Biophys J.

[138] Torres J, Parthasarathy K, Lin X, Saravanan R, Kukol A, Liu DX (2006). Model of a putative pore: the pentameric α-helical bundle of SARS coronavirus E protein in lipid bilayers. Biophys J.

[139] Torres J, Surya W, Li Y, Liu DX (2015). Protein-protein interactions of viroporins in coronaviruses and paramyxoviruses: new targets for antivirals?. Viruses..

[140] Surya W, Li Y, Verdià-Bàguena C, Aguilella VM, Torres J (2015). MERS coronavirus envelope protein has a single transmembrane domain that forms pentameric ion channels. Virus Res.

[141] Hsieh P-K, Chang SC, Huang C-C, Lee T-T, Hsiao C-W, Kou Y-H (2005). Assembly of severe acute respiratory syndrome coronavirus RNA packaging signal into virus-like particles is nucleocapsid dependent. J Virol.

[142] Tseng Y-T, Wang S-M, Huang K-J, Wang C-T (2014). SARS-CoV envelope protein palmitoylation or nucleocapsid association is not required for promoting virus-like particle production. J Biomed Sci.

[143] Maeda J, Maeda A, Makino S (1999). Release of coronavirus E protein in membrane vesicles from virus-infected cells and E protein-expressing cells. Virology..

[144] Tan Y-J, Fielding BC, Goh P-Y, Shen S, Tan TH, Lim SG (2004). Overexpression of 7a, a protein specifically encoded by the severe acute respiratory syndrome coronavirus, induces apoptosis via a caspase-dependent pathway. J Virol.

[145] Huang C, Ito N, Tseng C-TK, Makino S (2006). Severe acute respiratory syndrome coronavirus 7a accessory protein is a viral structural protein. J Virol.

[146] Tan Y-X, Tan TH, Lee MJ-R, Tham P-Y, Gunalan V, Druce J (2007). Induction of apoptosis by the severe acute respiratory syndrome coronavirus 7a protein is dependent on its interaction with the Bcl-XL protein. J Virol.

[147] Kanzawa N, Nishigaki K, Hayashi T, Ishii Y, Furukawa S, Niiro A (2006). Augmentation of chemokine production by severe acute respiratory syndrome coronavirus 3a/X1 and 7a/X4 proteins through NF-κB activation. FEBS Lett.

[148] Yuan X, Wu J, Shan Y, Yao Z, Dong B, Chen B (2006). SARS coronavirus 7a protein blocks cell cycle progression at G0/G1 phase via the cyclin D3/pRb pathway. Virology..

[149] Pan JA, Peng X, Gao Y, Li Z, Lu X, Chen Y (2008). Genome-wide analysis of protein-protein interactions and involvement of viral proteins in SARS-CoV replication. PLoS One.

[150] DeDiego ML, Pewe L, Alvarez E, Rejas MT, Perlman S, Enjuanes L (2008). Pathogenicity of severe acute respiratory coronavirus deletion mutants in hACE-2 transgenic mice. Virology..

[151] Yount B, Roberts RS, Sims AC, Deming D, Frieman MB, Sparks J (2005). Severe acute respiratory syndrome coronavirus group-specific open reading frames encode nonessential functions for replication in cell cultures and mice. J Virol.

[152] Schaecher SR, Touchette E, Schriewer J, Buller RM, Pekosz A (2007). Severe acute respiratory syndrome coronavirus gene 7 products contribute to virus-induced apoptosis. J Virol.

[153] Beale R, Wise H, Stuart A, Ravenhill BJ, Digard P, Randow F (2014). A LC3-interacting motif in the influenza a virus M2 protein is required to subvert autophagy and maintain virion stability. Cell Host Microbe.

[154] Subramani C, Nair VP, Anang S, Mandal SD, Pareek M, Kaushik N (2018). Host-Virus Protein Interaction Network Reveals the Involvement of Multiple Host Processes in the Life Cycle of Hepatitis E Virus. MSystems..

[155] Benga WJ, Krieger SE, Dimitrova M, Zeisel MB, Parnot M, Lupberger J (2010). Apolipoprotein E interacts with hepatitis C virus nonstructural protein 5A and determines assembly of infectious particles. Hepatology..

[156] Lu J, Qu Y, Liu Y, Jambusaria R, Han Z, Ruthel G (2013). Host IQGAP1 and Ebola virus VP40 interactions facilitate virus-like particle egress. J Virol.

[157] König R, Stertz S, Zhou Y, Inoue A, Hoffmann H-H, Bhattacharyya S (2010). Human host factors required for influenza virus replication. Nature..

[158] Börgeling Y, Schmolke M, Viemann D, Nordhoff C, Roth J, Ludwig S (2014). Inhibition of p38 mitogen-activated protein kinase impairs influenza virus-induced primary and secondary host gene responses and protects mice from lethal H5N1 infection. J Biol Chem.

[159] Ye Y, Hogue BG (2007). Role of the coronavirus E viroporin protein transmembrane domain in virus assembly. J Virol.

[160] Krijnse-Locker J, Ericsson M, Rottier P, Griffiths G (1994). Characterization of the budding compartment of mouse hepatitis virus: evidence that transport from the RER to the Golgi complex requires only one vesicular transport step. J Cell Biol.

[161] Tooze J, Tooze S (1985). Infection of AtT20 murine pituitary tumour cells by mouse hepatitis virus strain A59: virus budding is restricted to the Golgi region. Eur J Cell Biol.

[162] Arndt AL, Larson BJ, Hogue BG (2010). A conserved domain in the coronavirus membrane protein tail is important for virus assembly. J Virol.

[163] Nguyen V-P, Hogue BG (1997). Protein interactions during coronavirus assembly. J Virol.

[164] Ho Y, Lin P-H, Liu CY, Lee S-P, Chao Y-C (2004). Assembly of human severe acute respiratory syndrome coronavirus-like particles. Biochem Biophys Res Commun.

[165] Almazán F, DeDiego ML, Sola I, Zuñiga S, Nieto-Torres JL, Marquez-Jurado S (2013). Engineering a replication-competent, propagation-defective Middle East respiratory syndrome coronavirus as a vaccine candidate. MBio..

[166] DeDiego ML, Nieto-Torres JL, Jimenez-Guardeño JM, Regla-Nava JA, Castaño-Rodriguez C, Fernandez-Delgado R (2014). Coronavirus virulence genes with main focus on SARS-CoV envelope gene. Virus Res.

[167] Liu D, Inglis S (1991). Association of the infectious bronchitis virus 3c protein with the virion envelope. Virology..

[168] Yu X, Bi W, Weiss SR, Leibowitz JL (1994). Mouse hepatitis virus gene 5b protein is a new virion envelope protein. Virology..

[169] Locker JK, Griffiths G, Horzinek M, Rottier P (1992). O-glycosylation of the coronavirus M protein: differential localization of sialyltransferases in N-and O-linked glycosylation. J Biol Chem.

[170] Machamer CE, Mentone SA, Rose JK, Farquhar MG (1990). The E1 glycoprotein of an avian coronavirus is targeted to the cis Golgi complex. Proc Natl Acad Sci.

[171] Fischer F, Stegen CF, Masters PS, Samsonoff WA (1998). Analysis of constructed E gene mutants of mouse hepatitis virus confirms a pivotal role for E protein in coronavirus assembly. J Virol.

[172] Gosert R, Kanjanahaluethai A, Egger D, Bienz K, Baker SC (2002). RNA replication of mouse hepatitis virus takes place at double-membrane vesicles. J Virol.

[173] Goldsmith CS, Tatti KM, Ksiazek TG, Rollin PE, Comer JA, Lee WW (2004). Ultrastructural characterization of SARS coronavirus. Emerg Infect Dis.

[174] Snijder EJ, Van Der Meer Y, Zevenhoven-Dobbe J, Onderwater JJ, van der Meulen J, Koerten HK (2006). Ultrastructure and origin of membrane vesicles associated with the severe acute respiratory syndrome coronavirus replication complex. J Virol.

[175] Ulasli M, Verheije MH, de Haan CA, Reggiori F (2010). Qualitative and quantitative ultrastructural analysis of the membrane rearrangements induced by coronavirus. Cell Microbiol.

[176] Angelini MM, Akhlaghpour M, Neuman BW, Buchmeier MJ (2013). Severe acute respiratory syndrome coronavirus nonstructural proteins 3, 4, and 6 induce double-membrane vesicles. MBio..

[177] Hagemeijer Marne C., Monastyrska Iryna, Griffith Janice, van der Sluijs Peter, Voortman Jarno, van Bergen en Henegouwen Paul M., Vonk Annelotte M., Rottier Peter J.M., Reggiori Fulvio, de Haan Cornelis A.M. (2014). Membrane rearrangements mediated by coronavirus nonstructural proteins 3 and 4. Virology.

[178] Hagemeijer MC, Ulasli M, Vonk A, Reggiori F, Rottier PJ, de Haan CA (2011). Mobility and interactions of the coronavirus nonstructural protein 4. J Virol.

[179] Rossman JS, Lamb RA (2013). Viral membrane scission. Annu Rev Cell Dev Biol.

[180] Martyna A, Gómez-Llobregat J, Lindén M, Rossman JS (2016). Curvature Sensing by a Viral Scission Protein. Biochemistry..

[181] Roberts KL, Leser GP, Ma C, Lamb RA (2013). The amphipathic helix of influenza a virus M2 protein is required for filamentous bud formation and scission of filamentous and spherical particles. J Virol.

[182] Yuan B, Campbell S, Bacharach E, Rein A, Goff SP (2000). Infectivity of Moloney murine leukemia virus defective in late assembly events is restored by late assembly domains of other retroviruses. J Virol.

[183] Utley TJ, Ducharme NA, Varthakavi V, Shepherd BE, Santangelo PJ, Lindquist ME (2008). Respiratory syncytial virus uses a Vps4-independent budding mechanism controlled by Rab11-FIP2. Proc Natl Acad Sci.

[184] Rossman JS, Jing X, Leser GP, Lamb RA (2010). Influenza virus M2 protein mediates ESCRT-independent membrane scission. Cell..

[185] Parthasarathy K, Lu H, Surya W, Vararattanavech A, Pervushin K, Torres J (2012). Expression and purification of coronavirus envelope proteins using a modified β-barrel construct. Protein Expr Purif.

[186] Shen X, Xue J-H, Yu C-Y, Luo H-B, Qin L, Yu X-J (2003). Small envelope protein E of SARS: cloning, expression, purification, CD determination, and bioinformatics analysis. Acta Pharmacol Sin.

[187] Steinmann E, Penin F, Kallis S, Patel AH, Bartenschlager R, Pietschmann T (2007). Hepatitis C virus p7 protein is crucial for assembly and release of infectious virions. PLoS Pathog.

[188] Pinto LH, Lamb RA (2007). Controlling influenza virus replication by inhibiting its proton channel. Mol BioSyst.

[189] Takeda M, Pekosz A, Shuck K, Pinto LH, Lamb RA (2002). Influenza a virus M2 ion channel activity is essential for efficient replication in tissue culture. J Virol.

[190] Sakai A, Claire MS, Faulk K, Govindarajan S, Emerson SU, Purcell RH (2003). The p7 polypeptide of hepatitis C virus is critical for infectivity and contains functionally important genotype-specific sequences. Proc Natl Acad Sci.

[191] Jones CT, Murray CL, Eastman DK, Tassello J, Rice CM (2007). Hepatitis C virus p7 and NS2 proteins are essential for production of infectious virus. J Virol.

[192] Klimkait T, Strebel K, Hoggan MD, Martin MA, Orenstein J (1990). The human immunodeficiency virus type 1-specific protein vpu is required for efficient virus maturation and release. J Virol.

[193] Hsu K, Seharaseyon J, Dong P, Bour S, Marbán E (2004). Mutual functional destruction of HIV-1 Vpu and host TASK-1 channel. Mol Cell.

[194] Lazrak A, Iles KE, Liu G, Noah DL, Noah JW, Matalon S (2009). Influenza virus M2 protein inhibits epithelial sodium channels by increasing reactive oxygen species. FASEB J.

[195] Shimbo K, Brassard DL, Lamb RA, Pinto LH (1995). Viral and cellular small integral membrane proteins can modify ion channels endogenous to *Xenopus* oocytes. Biophys J.

[196] Song W, Liu G, Bosworth CA, Walker JR, Megaw GA, Lazrak A (2009). Respiratory syncytial virus inhibits lung epithelial Na^+^ channels by up-regulating inducible nitric-oxide synthase. J Biol Chem.

[197] Whitehead SS, Bukreyev A, Teng MN, Firestone C-Y, Claire MS, Elkins WR (1999). Recombinant respiratory syncytial virus bearing a deletion of either the NS2 or SH gene is attenuated in chimpanzees. J Virol.

[198] Wang K, Lu W, Chen J, Xie S, Shi H, Hsu H (2012). PEDV ORF3 encodes an ion channel protein and regulates virus production. FEBS Lett.

[199] Watanabe S, Watanabe T, Kawaoka Y (2009). Influenza A virus lacking M2 protein as a live attenuated vaccine. J Virol.

[200] Gladue DP, Holinka LG, Largo E, Sainza IF, Carrillo C, O'Donnell V (2012). Classical swine fever virus p7 protein is a viroporin involved in virulence in swine. J Virol.

[201] Pinto LH, Dieckmann GR, Gandhi CS, Papworth CG, Braman J, Shaughnessy MA (1997). A functionally defined model for the M2 proton channel of influenza a virus suggests a mechanism for its ion selectivity. Proc Natl Acad Sci.

[202] Agirre A, Barco A, Carrasco L, Nieva JL (2002). Viroporin-mediated membrane permeabilization pore formation by nonstructural poliovirus 2B protein. J Biol Chem.

[203] Grice A, Kerr I, Sansom M (1997). Ion channels formed by HIV-1 Vpu: a modelling and simulation study. FEBS Lett.

[204] Melton JV, Ewart GD, Weir RC, Board PG, Lee E, Gage PW (2002). Alphavirus 6K proteins form ion channels. J Biol Chem.

[205] Hyser JM, Estes MK (2015). Pathophysiological consequences of calcium-conducting viroporins. Annu Rev Virol.

[206] Gonzalez ME, Carrasco L (2003). Viroporins. FEBS Lett.

[207] Suzuki T, Orba Y, Okada Y, Sunden Y, Kimura T, Tanaka S (2010). The human polyoma JC virus agnoprotein acts as a viroporin. PLoS Pathog.

[208] Hyser JM, Collinson-Pautz MR, Utama B, Estes MK (2010). Rotavirus disrupts calcium homeostasis by NSP4 viroporin activity. MBio..

[209] Wang C, Takeuchi K, Pinto L, Lamb R (1993). Ion channel activity of influenza a virus M2 protein: characterization of the amantadine block. J Virol.

[210] Mould JA, Paterson RG, Takeda M, Ohigashi Y, Venkataraman P, Lamb RA (2003). Influenza B virus BM2 protein has ion channel activity that conducts protons across membranes. Dev Cell.

[211] Pham T, Perry JL, Dosey TL, Delcour AH, Hyser JM (2017). The rotavirus NSP4 viroporin domain is a calcium-conducting ion channel. Sci Rep.

[212] Premkumar A, Wilson L, Ewart G, Gage P (2004). Cation-selective ion channels formed by p7 of hepatitis C virus are blocked by hexamethylene amiloride. FEBS Lett.

[213] Zhang R, Wang K, Lv W, Yu W, Xie S, Xu K (2014). The ORF4a protein of human coronavirus 229E functions as a viroporin that regulates viral production. Biochim Biophys Acta.

[214] Li Y, Verdià-Baguena C, Dossena S, Surya W, Huang M, To J (2014). Inhibition of the human respiratory syncytial virus small hydrophobic protein and structural variations in a bicelle environment. J Virol.

[215] Schnell JR, Chou JJ (2008). Structure and mechanism of the M2 proton channel of influenza a virus. Nature..

[216] Hay A, Wolstenholme A, Skehel J, Smith MH (1985). The molecular basis of the specific anti-influenza action of amantadine. EMBO J.

[217] Wilson L, Mckinlay C, Gage P, Ewart G (2004). SARS coronavirus E protein forms cation-selective ion channels. Virology..

[218] Wilson L, Gage P, Ewart G (2006). Hexamethylene amiloride blocks E protein ion channels and inhibits coronavirus replication. Virology..

[219] Lee C, Yoo D (2005). Cysteine residues of the porcine reproductive and respiratory syndrome virus small envelope protein are non-essential for virus infectivity. J Gen Virol.

[220] Aguilella VM, Queralt-Martín M, Aguilella-Arzo M, Alcaraz A (2010). Insights on the permeability of wide protein channels: measurement and interpretation of ion selectivity. Integr Biol.

[221] Nieto-Torres JL, Verdiá-Báguena C, Jimenez-Guardeño JM, Regla-Nava JA, Castaño-Rodriguez C, Fernandez-Delgado R (2015). Severe acute respiratory syndrome coronavirus E protein transports calcium ions and activates the NLRP3 inflammasome. Virology..

[222] Surya W, Fung TS, Li Y, Verdia-Baguena C, Queralt-Martin M, To J (2017). Channel-inactivating mutations and their revertant mutants in the envelope protein of infectious bronchitis virus. J Virol.

[223] Hsu K, Han J, Shinlapawittayatorn K, Deschenes I, Marbán E (2010). Membrane potential depolarization as a triggering mechanism for Vpu-mediated HIV-1 release. Biophys J.

[224] Schubert U, Ferrer-Montiel AV, Oblatt-Montal M, Henklein P, Strebel K, Montal M (1996). Identification of an ion channel activity of the Vpu transmembrane domain and its involvement in the regulation of virus release from HIV-1-infected cells. FEBS Lett.

[225] van Kuppeveld FJ, Hoenderop JG, Smeets RL, Willems PH, Dijkman HB, Galama JM (1997). Coxsackievirus protein 2B modifies endoplasmic reticulum membrane and plasma membrane permeability and facilitates virus release. EMBO J.

[226] Wozniak AL, Griffin S, Rowlands D, Harris M, Yi M, Lemon SM (2010). Intracellular proton conductance of the hepatitis C virus p7 protein and its contribution to infectious virus production. PLoS Pathog.

[227] Westerbeck JW, Machamer CE (2018). The infectious bronchitis virus coronavirus envelope protein alters Golgi pH to protect spike protein and promote release of infectious virus. bioRxiv.

[228] Stevens Fred J., Argon Yair (1999). Protein folding in the ER. Seminars in Cell & Developmental Biology.

[229] Lim YX, Ng YL, Tam JP, Liu DX (2016). Human coronaviruses: a review of virus-host interactions. Diseases..

[230] Ron D, Walter P (2007). Signal integration in the endoplasmic reticulum unfolded protein response. Nat Rev Mol Cell Biol.

[231] Fung TS, Liu DX (2014). Coronavirus infection, ER stress, apoptosis and innate immunity. Front Microbiol.

[232] An S, Chen C-J, Yu X, Leibowitz JL, Makino S (1999). Induction of apoptosis in murine coronavirus-infected cultured cells and demonstration of E protein as an apoptosis inducer. J Virol.

[233] DeDiego ML, Nieto-Torres JL, Jiménez-Guardeño JM, Regla-Nava JA, Álvarez E, Oliveros JC (2011). Severe acute respiratory syndrome coronavirus envelope protein regulates cell stress response and apoptosis. PLoS Pathog.

[234] Nijmeijer S, Leurs R, Smit MJ, Vischer HF (2010). The Epstein-Barr virus-encoded G protein-coupled receptor BILF1 hetero-oligomerizes with human CXCR4, scavenges Gαi proteins, and constitutively impairs CXCR4 functioning. J Biol Chem.

[235] Moore ML, Chi MH, Luongo C, Lukacs NW, Polosukhin VV, Huckabee MM (2009). A chimeric A2 strain of respiratory syncytial virus (RSV) with the fusion protein of RSV strain line 19 exhibits enhanced viral load, mucus, and airway dysfunction. J Virol.

[236] Wei C, Ni C, Song T, Liu Y, Yang X, Zheng Z (2010). The hepatitis B virus X protein disrupts innate immunity by downregulating mitochondrial antiviral signaling protein. J Immunol.

[237] Tortorella D, Gewurz BE, Furman MH, Schust DJ, Ploegh HL (2000). Viral subversion of the immune system. Annu Rev Immunol.

[238] Cornell CT, Kiosses WB, Harkins S, Whitton JL (2007). Coxsackievirus B3 proteins directionally complement each other to downregulate surface major histocompatibility complex class I. J Virol.

[239] de Jong AS, Visch H-J, de Mattia F, van Dommelen MM, Swarts HG, Luyten T (2006). The coxsackievirus 2B protein increases efflux of ions from the endoplasmic reticulum and Golgi, thereby inhibiting protein trafficking through the Golgi. J Biol Chem.

[240] Ichinohe T, Pang IK, Iwasaki A (2010). Influenza virus activates inflammasomes via its intracellular M2 ion channel. Nat Immunol.

[241] Triantafilou K, Kar S, Vakakis E, Kotecha S, Triantafilou M (2013). Human respiratory syncytial virus viroporin SH: a viral recognition pathway used by the host to signal inflammasome activation. Thorax..

[242] Zhang K, Hou Q, Zhong Z, Li X, Chen H, Li W (2013). Porcine reproductive and respiratory syndrome virus activates inflammasomes of porcine alveolar macrophages via its small envelope protein E. Virology..

[243] Ito M, Yanagi Y, Ichinohe T (2012). Encephalomyocarditis virus viroporin 2B activates NLRP3 inflammasome. PLoS Pathog.

[244] Chan PK, Chan MC (2013). Tracing the SARS-coronavirus. J Thorac Dis.

[245] Bruning A, Aatola H, Toivola H, Ikonen N, Savolainen-Kopra C, Blomqvist S (2018). Rapid detection and monitoring of human coronavirus infections. New Microbes New Infect.

[246] Gretebeck LM, Subbarao K (2015). Animal models for SARS and MERS coronaviruses. Curr Opin Virol.

[247] CDC. About Coronaviruses: Prevention and Treatment 2017. Available from: https://www.cdc.gov/coronavirus/about/prevention.html.

[248] Zumla A, Chan JF, Azhar EI, Hui DS, Yuen K-Y (2016). Coronaviruses - drug discovery and therapeutic options. Nat Rev Drug Discov.

[249] Lamirande EW, DeDiego ML, Roberts A, Jackson JP, Alvarez E, Sheahan T (2008). A live attenuated severe acute respiratory syndrome coronavirus is immunogenic and efficacious in golden Syrian hamsters. J Virol.

[250] Fett C, DeDiego ML, Regla-Nava JA, Enjuanes L, Perlman S (2013). Complete protection against severe acute respiratory syndrome coronavirus-mediated lethal respiratory disease in aged mice by immunization with a mouse-adapted virus lacking E protein. J Virol.

[251] Saha A, Murakami M, Kumar P, Bajaj B, Sims K, Robertson ES (2009). Epstein-Barr virus nuclear antigen 3C augments Mdm2-mediated p53 ubiquitination and degradation by deubiquitinating Mdm2. J Virol.

[252] Tang H, Da L, Mao Y, Li Y, Li D, Xu Z (2009). Hepatitis B virus X protein sensitizes cells to starvation-induced autophagy via up-regulation of beclin 1 expression. Hepatology..

[253] Craik DJ, Fairlie DP, Liras S, Price D (2013). The future of peptide-based drugs. Chem Biol Drug Des.

[254] Wilson C, Arkin M, Vassilev L, Fry D (2010). Small-molecule inhibitors of IL-2/IL-2R: lessons learned and applied. Small-molecule inhibitors of protein-protein interactions.

[255] Newman DJ, Cragg GM (2012). Natural products as sources of new drugs over the 30 years from 1981 to 2010. J Nat Prod.

[256] Walensky LD, Bird GH (2014). Hydrocarbon-stapled peptides: principles, practice, and progress. J Med Chem.

[257] Bernal F, Tyler AF, Korsmeyer SJ, Walensky LD, Verdine GL (2007). Reactivation of the p53 tumor suppressor pathway by a stapled p53 peptide. J Am Chem Soc.

[258] Stewart ML, Fire E, Keating AE, Walensky LD (2010). The MCL-1 BH3 helix is an exclusive MCL-1 inhibitor and apoptosis sensitizer. Nat Chem Biol.

[259] Phillips C, Roberts LR, Schade M, Bazin R, Bent A, Davies NL (2011). Design and structure of stapled peptides binding to estrogen receptors. J Am Chem Soc.

[260] Zhang H, Zhao Q, Bhattacharya S, Waheed AA, Tong X, Hong A (2008). A cell-penetrating helical peptide as a potential HIV-1 inhibitor. J Mol Biol.

[261] Jamieson A, Robertson N (2015). Regulation of protein-protein interactions using stapled peptides. Rep Org Chem.

[262] Cui H-K, Qing J, Guo Y, Wang Y-J, Cui L-J, He T-H (2013). Stapled peptide-based membrane fusion inhibitors of hepatitis C virus. Bioorg Med Chem.

[263] Gaillard V, Galloux M, Garcin D, Eléouët J-F, Le Goffic R, Larcher T (2017). A short double-stapled peptide inhibits respiratory syncytial virus entry and spreading. Antimicrob Agents Chemother.

[264] Zhang H, Curreli F, Waheed AA, Mercredi PY, Mehta M, Bhargava P (2013). Dual-acting stapled peptides target both HIV-1 entry and assembly. Retrovirology..

[265] Han J, Cong X (2018). The stapled peptides derived from hepatitis B virus core protein hijack viral replication. J Hepatol.

[266] Kaspar AA, Reichert JM (2013). Future directions for peptide therapeutics development. Drug Discov Today.

[267] Cromm PM, Spiegel J, Grossmann TN (2015). Hydrocarbon stapled peptides as modulators of biological function. ACS Chem Biol.

[268] Klionsky DJ (2007). Autophagy: from phenomenology to molecular understanding in less than a decade. Nat Rev Mol Cell Biol.

[269] Paul P., Münz C. (2016). Autophagy and Mammalian Viruses. Advances in Virus Research.

[270] Jackson WT (2015). Viruses and the autophagy pathway. Virology..

[271] Joubert P-E, Werneke SW, de la Calle C, Guivel-Benhassine F, Giodini A, Peduto L (2012). Chikungunya virus-induced autophagy delays caspase-dependent cell death. J Exp Med.

[272] Orvedahl A, MacPherson S, Sumpter R, Tallóczy Z, Zou Z, Levine B (2010). Autophagy protects against Sindbis virus infection of the central nervous system. Cell Host Microbe.

[273] Orvedahl A, Alexander D, Tallóczy Z, Sun Q, Wei Y, Zhang W (2007). HSV-1 ICP34. 5 confers neurovirulence by targeting the Beclin 1 autophagy protein. Cell Host Microbe.

[274] Gannagé M, Dormann D, Albrecht R, Dengjel J, Torossi T, Rämer PC (2009). Matrix protein 2 of influenza a virus blocks autophagosome fusion with lysosomes. Cell Host Microbe.

[275] Tallóczy Z, Virgin I, Herbert LB (2006). PKR-dependent xenophagic degradation of herpes simplex virus type 1. Autophagy..

[276] Kyei GB, Dinkins C, Davis AS, Roberts E, Singh SB, Dong C (2009). Autophagy pathway intersects with HIV-1 biosynthesis and regulates viral yields in macrophages. J Cell Biol.

[277] Dreux M, Gastaminza P, Wieland SF, Chisari FV (2009). The autophagy machinery is required to initiate hepatitis C virus replication. Proc Natl Acad Sci.

[278] Wong J, Zhang J, Si X, Gao G, Mao I, McManus BM (2008). Autophagosome supports coxsackievirus B3 replication in host cells. J Virol.

[279] Guo L, Yu H, Gu W, Luo X, Li R, Zhang J (2016). Autophagy negatively regulates transmissible gastroenteritis virus replication. Sci Rep.

[280] Sun M-X, Huang L, Wang R, Yu Y-L, Li C, Li P-P (2012). Porcine reproductive and respiratory syndrome virus induces autophagy to promote virus replication. Autophagy..

[281] Prentice E, Jerome WG, Yoshimori T, Mizushima N, Denison MR (2004). Coronavirus replication complex formation utilizes components of cellular autophagy. J Biol Chem.

[282] Ao D, Guo H-C, Sun S-Q, Sun D-H, Fung TS, Wei Y-Q (2015). Viroporin activity of the foot-and-mouth disease virus non-structural 2B protein. PLoS One.

[283] Crawford SE, Hyser JM, Utama B, Estes MK (2012). Autophagy hijacked through viroporin-activated calcium/calmodulin-dependent kinase kinase-β signaling is required for rotavirus replication. Proc Natl Acad Sci.

[284] Liu B, Panda D, Mendez-Rios JD, Ganesan S, Wyatt LS, Moss B (2018). Identification of Poxvirus Genome Uncoating and DNA Replication Factors with Mutually Redundant Roles. J Virol.

[285] Castaño-Rodriguez C, Honrubia JM, Gutiérrez-Álvarez J, DeDiego ML, Nieto-Torres JL, Jimenez-Guardeño JM (2018). Role of severe acute respiratory syndrome coronavirus Viroporins E, 3a, and 8a in replication and pathogenesis. mBio..

[286] Chen I-Y, Moriyama M, Chang M-F, Ichinohe T (2019). Severe acute respiratory syndrome coronavirus viroporin 3a activates the NLRP3 inflammasome. Front Microbiol.

